# A successive time-to-event model of phyllochron dynamics for hypothesis testing: application to the analysis of genetic and environmental effects in maize

**DOI:** 10.1186/s13007-023-01029-7

**Published:** 2023-06-07

**Authors:** Sandra Plancade, Elodie Marchadier, Sylvie Huet, Adrienne Ressayre, Camille Noûs, Christine Dillmann

**Affiliations:** 1 UR MIAT, University of Toulouse, INRAE, 31320 Castanet-Tolosan, France; 2 GQE - Le Moulon, Université Paris-Saclay, INRAE, CNRS, AgroParisTech, IDEEV, 12 route 128, 91190 Gif-sur-Yvette, France; 3 MaIAGE, Université Paris-Saclay, INRAE, 78350 Jouy-en-Josas, France; 4 Cogitamus Laboratory, 31320 Castanet-Tolosan, France

**Keywords:** Climate variables, Divergent selection, Genotypic effects, Hypothesis testing model, Maize, Phenology and plant development, Phyllochron, Stochastic process, Time to event

## Abstract

**Background:**

The time between the appearance of successive leaves, or phyllochron, characterizes the vegetative development of annual plants. Hypothesis testing models, which allow the comparison of phyllochrons between genetic groups and/or environmental conditions, are usually based on regression of thermal time on the number of leaves; most of the time a constant leaf appearance rate is assumed. However regression models ignore auto-correlation of the leaf number process and may lead to biased testing procedures. Moreover, the hypothesis of constant leaf appearance rate may be too restrictive.

**Methods:**

We propose a stochastic process model in which emergence of new leaves is considered to result from successive time-to-events. This model provides a flexible and more accurate modeling as well as unbiased testing procedures. It was applied to an original maize dataset collected in the field over three years on plants originating from two divergent selection experiments for flowering time in two maize inbred lines.

**Results and conclusion:**

We showed that the main differences in phyllochron were not observed between selection populations but rather between ancestral lines, years of experimentation and leaf ranks. Our results highlight a strong departure from the assumption of a constant leaf appearance rate over a season which could be related to climate variations, even if the impact of individual climate variables could not be clearly determined.

**Supplementary Information:**

The online version contains supplementary material available at 10.1186/s13007-023-01029-7.

## Introduction

In annual plant species, growth and development are critical in determining the length of the life cycle (from germination to seed maturation), which in turn has an impact on biomass production. Plant development is characterized by the repeated production and growth of different organs that in the early stages are often protected by other tissues. Measuring the time between the appearance of successive leaves or *phyllochron* is a widely used non-destructive method to determine the overall pace of plant development. As plant growth rates vary with temperature, the time-scale is usually converted to accumulated thermal time (ATT) measured in degree-days [1]. The objective of this transformation is to propose a new variable that captures the component of plant growth rate variation that is independent of the average thermal time [2].

Two classes of statistical models for investigating plant development can be found in the literature: predictive models and hypothesis-testing models. The objective of predictive models is to simulate crop development, either at the field level using compartment models, or at the plant level allowing for the description of plant architecture. Compartment models are based on differential rate equations that simulate how a plant community at the field scale responds to the environment (e.g. APSIM [3–6]); Functional Structural Plant Models (FSPMs) integrate organ development in individual plants and the study of plant interactions in the field [7–9]. These models are developed for predictive purposes, e.g. to predict biomass production in different genotypes under various climate scenarios, sowing densities or management practices. The number of parameters is generally high and sensitivity analyses may be conducted to enhance interpretation. Predictions are generally valid under a restricted range of conditions (genotypes, environments).

Hypothesis-testing models compare parameters between classes (e.g. environmental conditions, genotypic groups) and test the statistical significance of the condition effect. In this paper, we are interested in hypothesis-testing models of phyllochron. Existing models are based on a regression of ATT on observed leaf numbers with independent errors, usually with an assumption of linear relationship. Unlike regression models which can be applied either to continuous or discrete longitudinal phenotypic traits, we propose to use the specificity of phyllochron data to model the leaf appearance process as resulting from successive events, namely the appearance of successive leaves, and address some of the limitations of the existing models.

**Fig. 1:**
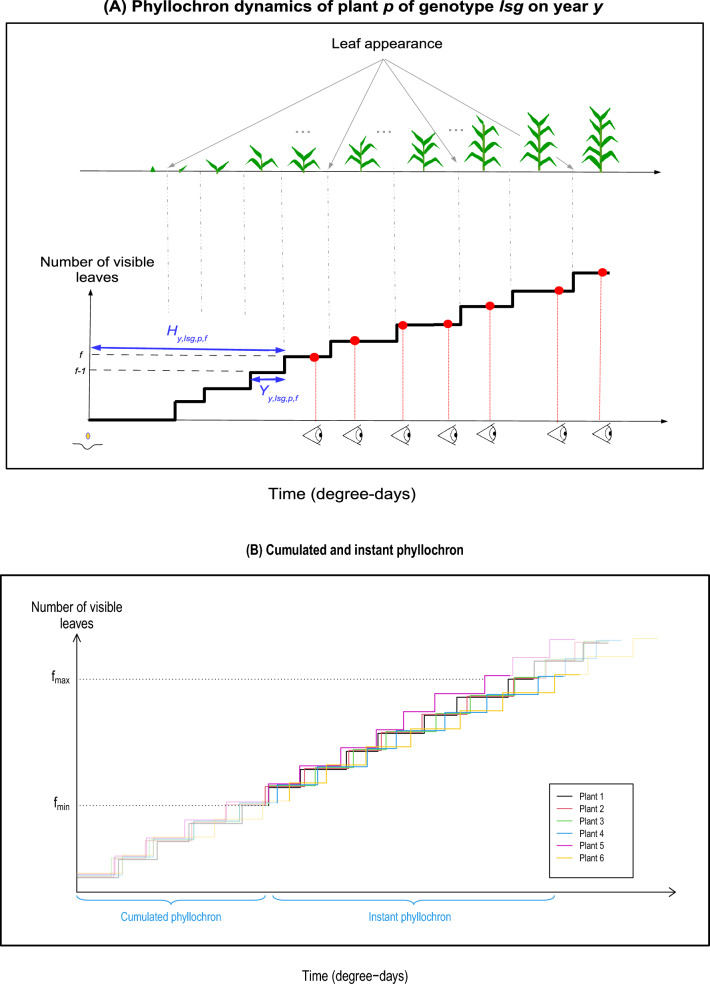
Illustration of the phyllochron process. **A** Phyllochron dynamics (process) in plant *p* belonging to genotype *lsg*. The step curve corresponds to the number of visible leaves over time; the interval between steps corresponds to the time interval between successive leaves denoted $Y_{y,lsg,p,f}$; the time between sowing and the appearance of leaf *f* is denoted $H_{y,lsg,p,f}$, and is equal to the sum of the time intervals between leaves until rank *f*. Red dots correspond to the time of measurement, when the number of leaves is recorded. **B** Simulated phyllochron dynamics for six plants from the same genotype (one color per plant). For each plant, the light-colored curve corresponds to phyllochrons over the entire process of leaf appearance, while the dark-colored curve corresponds to phyllochrons for the restricted range of leaf ranks $[f_{\min }, f_{\max }]$ imposed by experimental constraints. The first modeled leaf $f_{\min }$ determines the boundary between the *cumulated phyllochron*, which corresponds to the time between sowing and appearance of leaf $f_{\min }$, and the *instant phyllochron* which corresponds to all intervals between successive leaves within the $[f_{\min }, f_{\max }]$ range

The most frequently used hypothesis-testing model is the *linear model* which assumes a linear leaf appearance process in thermal time, or equivalently a constant rate of leaf appearance, inferred by a linear regression of the times of measurement (in ATT) on the observed number of leaves, for each plant separately [10–12]. More precisely, the phyllochron of each plant is summarized by a single value, and the difference in phyllochron between different conditions is tested using a parametric or a non-parametric univariate test (e.g. F-test, Mann–Whitney) or within a linear mixed model for more complex designs. This class of models suffers two limitations: (i) the assumption of a constant leaf appearance rate and (ii) the underlying assumptions related to the regression model. Assumption (i) may be too restrictive since growth parameters have been shown to be affected by within-season variations of environmental variables [13] and to be independent between leaf ranks [14]. Some more flexible models for the relationship between number of leaves and ATT have been proposed, in particular bi- or tri-linear regression for rice and maize [15, 16] or spline regression for wheat [17], but without a statistical procedure to compare the phyllochron of groups or conditions. Limitation (ii) is less obvious. The statistical flaws of regression models for hypothesis testing on time processes have been pointed over the past decade [18, 19], notably for the study of seed germination, but the higher complexity of survival analysis methods compared to regression models may explain why the former is not commonly used. Phyllochron analyses based on regression models also suffer from similar flaws, being simultaneously statistically biased and based on a unrealistic modeling of the plant level variations. Indeed, regression models implicitly assume that the leaf appearance process is the sum of a general trend and of independent centered random variations, which results in a non-increasing process. More generally, the assumption of independent random variations is unrealistic, since the phyllochron process is by nature auto-correlated i.e. the number of leaves at time $t' > t$ depends on the number of leaves at time *t*. Notably, this phenomenon results in a higher variability of leaf appearance time as the leaf rank increases, which can be observed for instance in Fig. 1 in [20] and [14]. But under regression models, variations of the leaf number process around a mean value, either the phyllochron coefficient for the linear model or the mean temporal trend for more flexible models, can be either negative or positive, so the leaf appearance process is not increasing. This phenomenon is illustrated in Fig. 1 and the conceptual differences between time-to-events and regression models is detailed in the first subsection of the “Results” section. Besides, in the linear model, where the plant level phyllochron comes down to a regression slope computed prior to the statistical analysis, the statistical power is independent of the number of time points for monitoring, which is intuitively and statistically flawed. A regression slope model implemented directly on the observations could circumvent this limitation, but is not existing in the literature, to the best of our knowledge. Moreover, the limitations of regression models would still hold for slope regression models.

Thus, our phyllochron model based on successive time-to-events aims to address the shortcomings of regression models by combining (i) a flexible phyllochron structure, (ii) a more realistic modeling of the variations and (iii) an accurate testing procedure in the simple framework of class comparison. It is comparable to recent models that were developed for a single time-to-event outcome [21, 22] which are based on survival analysis, a class of methods widely used in the medical field but still rarely applied in agriculture and specifically geared toward time-to-event variables.

To assess the benefit of our approach, we made use of original plant material produced from two divergent selection experiments (DSEs) for flowering time conducted over 12 years within two maize inbred lines under agronomic conditions [23–25]. Within homogeneous genetic backgrounds, phenological shifts between Early and Late progenitors were selected, resulting in a difference of up to three weeks after 15 generations (150 degree-days [24, 25]). Representative progenitors from generation G13 were chosen to monitor plant growth over three years, from 2014 to 2016.

In addition to looking for differences in phyllochron between genotypes of the G13 generation, we investigated whether the time varying climate conditions experienced during growth could affect the phyllochron. To address this question, a modeling procedure that allows changes in leaf appearance rate was required.

Although the ATT is supposed to account for the thermal dependency of growth rates, other climate variables have been shown to modulate developmental rates in maize [20]. Notably, phyllochron seems impacted by photosynthetically active radiation: previous studies showed that the rate of leaf appearance increases with photoperiod or irradiance [26, 27] while [16] recently showed that phyllochron and radiation are positively correlated. In wheat, long photoperiods also increase the leaf appearance rate [28] whereas nitrogen stress has been shown to decrease this rate [29]. In maize, sowing date and mixed stands culture with wheat are other factors affecting the leaf appearance rate, which can be considered as the resulting effect of multiple climate variables [27, 30].

In this paper, we take advantage of a novel approach for phyllochron modeling to implement a procedure that accounts for experimental constraints, together with a testing procedure. In addition, a two-step procedure is proposed to assess the impact of climate variables on departures from a constant leaf appearance rate.

## Materials and methods

### Experimental design and data collection

#### Plant material

Plants used in the experiments were obtained from 12 years of divergent selection for flowering time in two maize inbred lines MBS847 (MBS) and F252 hereafter called *ancestral lines*. Ancestral seed lots of each line were used to produce two *selection populations*: one Early and one Late. Within each selection population, the earliest and latest flowering plants were repeatedly selected and self-fertilized. At generation G13, each selection population comprised two or three *genotypes*. A genotype is a set of plants derived by selfing without selection from a single plant that was selected at G13. Note that each selected plant at G13 is derived by selfing and selection from a single plant from the ancestral seed lot (G0). For F252, early genotypes were named FE036 and FE039 and late genotypes FL027, FL317 and FL318. For MBS, early genotypes were named ME049 and ME052 and late genotypes ML040 and ML053. The selection and selfing processes is described in details in [23]. The hierarchical structure of the plant material is summarized in Table 1. Contrasted flowering times were observed in G13 plants from the two ancestral lines, as well as between Early and Late selection populations from both ancestral lines [24, 25].

#### Crop experiment

G13 plants from the nine genotypes were sown in 2014, 2015 and 2016 at the Saclay Plateau (France) following a random row design. Each row (5.2 m in length) contained 25 equally spaced maize seeds. The distance between two rows was 0.8 m. Rows were spatially arranged into 16-row wide rectangular plots with two control rows at coordinates *X*1 and *X*16. Each ancestral line was sown on a spearate plot. In 2014, planting consisted of three rows per genotype; In 2015, three to five rows per genotype; In 2016, 12 rows per genotype although only ME052 and ML040 from the ancestral line MBS were assessed. To avoid seedling predation by birds, experimental plots were protected with netting immediately after sowing until most plants had at least six to seven visible leaves. After removing the netting, the number of leaves was counted on each plant twice a week (every 2–4 days) corresponding to the time interval between stages GRO:0007013 and GRO:0007015 following the Cereal Plant Development Ontology (https://bioportal.bioontology.org/ontologies/GRO-CPD), until the emergence of panicle. The emergence of the tip of a leaf was considered to be the criterion for leaf appearance. To avoid assessment errors due to leaf degradation, ranks of the third leaf and of every odd new leaf after the netting was removed were marked on the leaf with a pencil (requiring a brief removal of the netting for rank three).

Some plants in the study were dissected for further analysis, around a median leaf rank of 8.5 (year 2014), 10 (year 2015) and 9 (year 2016). These partially observed plants were used to infer the phyllochron model but their contribution was limited to the estimation of the apperances of the first leaves.

Altogether, we collected data from 1795 plants. For each plant, the leaf rank of the youngest visible leaf was recorded at various time points. The appearance of the last two leaves was not modeled and corresponding data were not included in the analysis. Note that the actual time of appearance of each leaf, which characterizes the phyllochron, was not observed; data here pertain to the time interval in which each leaf appears. Because of measurement errors, some plants displayed a decreasing number of leaves between two successive time points; these plants were discarded from the final data set. In 2014, complete phyllochron data were collected from 318 plants over 8–17 [mean 12.5] observation time points. In 2015, complete phyllochron data were obtained from 371 plants over 8–21 [mean 13] time points, and partial measurements were obtained from 328 plants. In 2016, complete phyllochron data were obtained from 196 plants over 6–15 [mean 11.2] time points, and partial measurements were obtained from 233 plants.

#### Climate variables

Data for climate variables were extracted from hourly records from the climate station located near the field sites, and downloaded from INRAE’s climatik database [31]. Climate variables are described in Table 2. Mean daytime temperature was used to compute the thermal time with the parameters estimated in [1] following the method described in [32].

### Statistical model of phyllochron

The phyllochron of a plant is characterized by the time between the appearance of successive leaves, or equivalently the time when a new leaf appears. When measurements are made at set times, these values cannot be observed (Fig. 1a). Thus, we defined a statistical phyllochron model that, combined with an inference algorithm, enabled us to estimate the mean phyllochron at the genotype level. We denote by subscripts *lsg* the $g{\textrm{th}}$ genotype from selection population *s* (Early or Late) derived from ancestral line *l* (F252 or MBS). This phyllochron model was implemented separately for each year of the experiment. All notations are summarized in Table 3.

#### Statistical model over the entire phyllochron

Let $Y_{y,\,lsg,\,p,\,f}$ be the unobserved time length between the appearance of leaves of rank $(f-1)$ and *f* on plant *p* from genotype *lsg* in year *y*. The phyllochron of plant *p* is characterized by the vector $(Y_{y,\,lsg,\,p,\,f})_{f =1, \ldots , F^{lsg}}$ with $F^{lsg}$ the overall maximum number of leaves for genotype *lsg*. Then, the time of appearance of leaf *f* on plant *p* from genotype *lsg* is

$$\begin{aligned} H_{y,\,lsg,\,p,\,f} = \sum _{f'=1}^{f} Y_{y,\,lsg,\,p,\,f'}. \end{aligned}$$

The variables are illustrated in Fig. 1a. We assume that the time between the appearance of successive leaves $Y_{y,\,lsg,\,p,\,f}$ depends on the plant genotype *lsg* and on the leaf rank $f =1, \ldots , F^{lsg}$:

1 $$\begin{aligned} Y_{y,\,lsg,\,p,\,f}= \mu _{y,\,lsg,\,f} + \text {plant level variation} \end{aligned}$$

Thus, the mean phyllochron of genotype *lsg* on year *y* is characterized by the vector ${\varvec{\mu }}_{y,\,lsg} = (\mu _{y,\,lsg,\,f})_{f=1, \ldots , F^{lsg} }$, and ${\overline{\mu }}_{y,\,lsg,\,f}= \sum _{f'=1}^f \mu _{y,\,lsg,\,f'}$ corresponds to the mean time of appearance of leaf *f* for plants of genotype (*y*, *lsg*). Note that no parametric form is assumed for the variations of the genotype level phyllochron along leaf ranks, namely the function $f \mapsto \mu _{y,\,lsg,\,f}$. Moreover, the time lengths between the appearance of successive leaves on each plant are assumed to be independent Gaussian distributed variables, thus

2 $$\begin{aligned} {{\textbf {Y}}}_{y,lsg,p}= (Y_{y,\,lsg,\,p,\,f})_{f=1, \ldots , F^{lsg}} \sim {\mathcal {N}}_{F^{lsg}}(\mu _{y,\,lsg}, \Sigma _{y,\,lsg}) \end{aligned}$$

with $\Sigma _{y,\,lsg}$ the diagonal variance covariance matrix with a vector of diagonal terms $(\sigma _{y,\,lsg,\,f}^2)_{f=1 \dots , F^{lsg}}$. The assumption of normal distribution originates from constraints on the numerical implementation, since the algorithm requires tools that are specific to Gaussian distributions (see the “Parameter inference” section and Additional file 1: A). The main parameters of interest are $\left( \mu _{y,\,lsg,\,f}\right) _f$, while $\left( \sigma _{y,\,lsg,\,f}\right) _f$ may be considered to be nuisance parameters. Note that the leaf appearance times $(H_{y,\,lsg,\,p,\,1})_{f=1, \ldots , F^{lsg}}$ on a plant *p* are not independent as they result from the accumulation of independent variables corresponding to the times between the appearance of successive leaves $(Y_{y,\,lsg,\,p,\,f})_f$.

#### Taking into account experimental constraints: a restricted range of leaf ranks

Observations in the field started at a fixed time point, resulting in variations in the first leaf rank observed between plants. Thus to increase the precision of parameter estimates for each genotype *lsg*, we modeled the phyllochron on a range of leaf ranks $[f_{\min }^{y,\,lsg},f_{\max }^{y,\,lsg}]$ such that leaf ranks $f_{\min }^{y,\,lsg}-1$ and $f_{\max }^{y,\,lsg}$ were observed on at least 10 plants from this genotype in year *y*.

We found different leaf rank intervals for different genotypes. This difference may be due to both statistical sampling and biological variation. Nevertheless, some analyses required a common leaf rank interval, so we also considered $[f^0_{\min }, f^0_{\max }] = \bigcap _{y,\,lsg} [f_{\min }^{y,\,lsg},f_{\max }^{y,\,lsg}]=[8,13]$. However, information is lost in such a restricted interval. Therefore, comparative analyses between genotypes were performed using the common interval, while the temporal trends in phyllochron dynamics as well as the influence of climate were studied on the genotype-specific intervals $[f_{\min }^{y,\,lsg},f_{\max }^{y,\,lsg}]$.

#### Statistical model on the restricted leaf rank interval: cumulated and instant phyllochron

First leaves being not or rarely observed, the corresponding parameters $(\mu _{y,\,lsg,\,f}, \sigma _{y,\,lsg,\,f})$ could not be estimated. Thus, we defined a cumulated phyllochron which is the period of time spanning the appearance of more than one leaf. More precisely, for a leaf rank interval $[f_{\min }, f_{\max }]$, which is equal to either the leaf rank range for all genotypes $[f_{\min }^0, f_{\max }^0]$ or to the genotype specific leaf rank range $[f_{\min }^{y,\,lsg}, f_{\max }^{y,\,lsg}]$, we can infer the following distributions (Fig. 1b):

- The distribution of $(Y_{lsg,p,f }\sim {\mathcal {N}}(\mu _{y,\,lsg,\,f}, \sigma ^2_{y,\,lsg,\,f}))_{f=f_{\min }+1, \ldots ,f_{\max }}$, that we denote *instant phyllochron*.
- The distribution of $H_{y,\,lsg,\,p,\,f_{\min }} \sim {\mathcal {N}} \left( \mu ^{C(f_{\min })}_{y,\,lsg}, \left( \sigma ^{C(f_{\min })}_{y,\,lsg} \right) ^2 \right)$ with
  $$\begin{aligned} \mu ^{C(f_{\min })}_{y,\,lsg} = \sum _{f'=1}^{f_{\min }} \mu _{y,\,lsg,\,f'}, \quad \sigma ^{C(f_{\min })}_{y,\,lsg} = \sqrt{\sum _{f'=1}^{f_{\min }} \sigma ^2_{y,\,lsg,\,f'}} \end{aligned}$$
  that we denote *cumulated phyllochron*.

Subscript $C(f_{\min })$ indicates the highest leaf rank used to calculate the cumulated phyllochron. This value is the result of the plant development since sowing. Note that the distinction between cumulated and instant phyllochron results from $f_{\min }$ which depends on the experimental conditions: if recording starts later in development, the cumulated phyllochron will be the resultant of more developmental stages.

#### Parameter inference

**Fig. 2:**
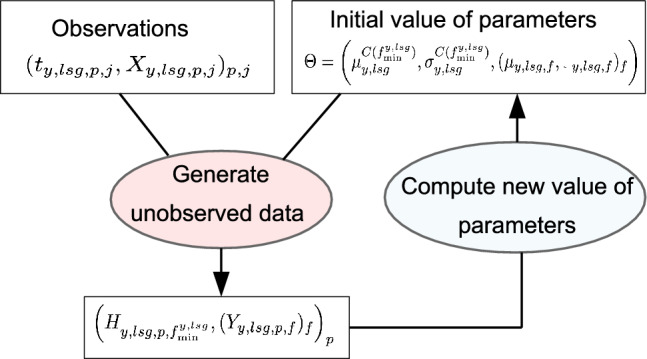
Monte Carlo Expectation Maximization algorithm. Starting from an initial value of the parameters, the unobserved times of leaf appearance are drawn from their distribution given the observed data. Then, the maximum likelihood estimator is inferred from the simulated (unobserved) data, producing a new estimate of the parameters. The algorithm is iterated until stabilization of the parameters

For each genotype, the parameters

$$\begin{aligned} \left( \mu ^{C\left(f_{\min }^{y,\,lsg}\right)}_{y,\,lsg},\,\sigma ^{C\left(f_{\min }^{y,\,lsg}\right)}_{y,\,lsg},\, \left( \mu _{y,\,lsg,\,f},\, \sigma _{y,\,lsg,\,f}\right)_{f=f_{\min }^{y,\,lsg}+1, \ldots ,f_{\max }^{y,\,lsg}}\right) \end{aligned}$$

were estimated via a Monte Carlo Expectation Maximization (MCEM) algorithm [33] adapted to latent variable models (Fig. 2 and Additional file 1: A). In our context, the latent variables are the (unobserved) times of leaf appearance of all plants, and the observations are restricted to time intervals in which each leaf appears on each plant. The MCEM algorithm requires sampling from the distribution of the latent variables given the observations, which therefore correspond to a truncated multivariate distribution. Although the computation time of classic rejection methods dramatically increases with the dimension, efficient tools have been developed for the multivariate normal distribution [34]. The parameters of the cumulated phyllochron for the restricted interval $[f_{\min }^0, f_{\max }^0] \subset [f_{\min }^{y,\,lsg}, f_{\max }^{y,\,lsg}]$ can be directly deduced. Indeed, if $f^0 > f_{\min }^{y,\,lsg}$:

$$\begin{aligned} \mu ^{C\left(f_{\min }^0\right)}_{y,\,lsg}&= \mu ^{C\left(f_{\min }^{y,\,lsg}\right)}_{y,\,lsg} + \sum _{f=f_{\min }^{y,\,lsg}+1} ^{f^0_{\min }}\mu _{y,\,lsg,\,f} \\ \left( \sigma ^{C\left(f_{\min }^0\right)}_{y,\,lsg} \right) ^2&= \left( \sigma ^{C\left(f_{\min }^{y,\,lsg}\right)}_{y,\,lsg}\right) ^2 + \sum _{f=f_{\min }^{y,\,lsg}+1} ^{f^0_{\min }}\left( \sigma _{y,\,lsg,\,f} \right) ^2 \end{aligned}$$

### Model comparisons

#### Tests of genotypic groups effects

We made use of the hierarchical groupings of the plants studied to understand the genetic factors that impact phyllochron values. Indeed, differences between plants may come from ancestral lines (F252 *vs* MBS), selection populations (Early *vs* Late), or genotypes within a selection population from an ancestral line (see Table 1 and the “Plant material” in “Materials and methods” section). Statistical differences can occur either before the first observations and target the $(\mu ^C, \sigma ^C)$ parameters, and/or on the modeled leaf ranks and target the $(\mu , \sigma )$ parameters. We ran seven different models $M_{i,j}$ (Table 4-A) on the leaf rank range common to all genotypes $[f_{\min }^0, f_{\max }^0]$, the *i* (resp. *j*) indices denoting the level of dependence of the cumulated (resp. instant) phyllochron. *i* (resp. *j*) values indicate that the cumulated (resp. instant) phyllochron is considered to be identical across all plants (0), within an ancestral line (1), within a selection population (2) or within a genotype (3).

Model $M_{00}$ assumes that all plants have the same phyllochron distribution irrespective of the ancestral line, selection population or genotype. Models $M_{10}$ and $M_{11}$ suppose that the phyllochron varies with the ancestral line for at least one leaf rank, either during early developmental stages ($M_{10}$) or at any point in the season ($M_{11}$); $M_{11}$ is preferred to $M_{10}$ when the phyllochron differences occur between observed leaf ranks. Similarly, models $M_{21}$ and $M_{22}$ assume differences in phyllochron between Early and Late populations for at least one leaf rank, while models $M_{32}$ and $M_{33}$ assume differences between genotypes.

As the phyllochron estimates highlighted obvious differences between years, a separate analysis was run for each year. Also, comparisons between $M_{12}$/$M_{22}$ and $M_{11}$ were performed either by pooling the two ancestral lines, or within each ancestral line. Comparisons between $M_{23}$/$M_{33}$ and $M_{22}$ were also performed within ancestral line and selection population.

Note that because of the hierarchical structure of the models, significant differences between genotypes may result in significant differences between selection populations, independently of a divergent selection effect.

#### Permutation test for model comparison

Comparison of pairs of nested models was based on the likelihood ratio test (LRT) statistic. In the case of independent observations, the LRT statistic under the more parsimonious model would be asymptotically distributed as a $\chi ^2$ whose degree of freedom is the difference of number of parameters between the two models. The $\chi ^2$-LRT may be biased due to the finite sample size and to the correlation between plants from the same row. Therefore, for each comparison, the distribution under the parsimonious model was obtained by repeated permutations of the plant rows between groups. This permutation test was implemented only for those comparisons for which the number of possible permutations was larger than 20.

#### Parametric sub-models of phyllochron dynamics

In order to identify trends in the departure from the classic constant phyllochron model, we considered parametric sub-models of instant phyllochron as a function of leaf rank that can capture the general trends by summarizing the vector $(\mu _{y,\,lsg,\,f})_{f_{\min }^{lsg}+1, \ldots , f_{\max }^{lsg}}$ from a reduced set of parameters for each genotype and year. We considered four parametric models: (i) the constant model supposes that the phyllochron is constant throughout the season, (ii) the constant-rate model supposes a constant increase or decrease of the phyllochron with leaf rank, (iii) the piecewise constant model allows a single change in the phyllochron at a given leaf stage, (iv) the piecewise linear model allows two phases where the phyllochron can increase or decrease. Among the models that are significantly more accurate than the constant model ($\chi ^2$-LRT, $p<0.01$), we selected the one with the lowest AIC (details in Additional file 1: B). The $\chi ^2$-LRT extracts the models that are significantly more accurate than the classic constant model, then the AIC enables to select among these non-nested models without considering any of them as a reference.

### Impact of climate on the phyllochron

In order to assess the impact of climate on the variations of phyllochron within a growing season and between years, we considered a model where the time intervals between successive leaves were regressed over a function of longitudinal climate variables prior to leaf appearance.

#### General model

Our climate model is defined on a calendar time scale. Therefore, phyllochron parameters were first back-transformed into their calendar time equivalent. Thus $\mu ^{cal}_{y,\,lsg,\,f}$ and $\mu ^{C(f),cal}_{y,\,lsg}$ denote in calendar time the genotype level mean time between the appearance of leaf $(f-1)$ and leaf *f* and the mean appearance time of leaf *f* since sowing in genotype *lsg* and year *y*. Then, for year *y*, genotype *lsg* and leaf rank *f*, we considered the general model:

3 $$\begin{aligned} \mu _{y,\,lsg,\,f}=\alpha _{y,\,l} + \beta _{lsg} + \sum _{\text {c climatic variable} } \varphi \left( \{ X_{y,\,c} (\mu ^{C(f),cal}_{y,\,lsg} - t \}_{t \ge 1} | \gamma _{y,\,l,\,c} \right) + \varepsilon _{y,\,lsg,\,f} \end{aligned}$$

where $\{ X_{y,\,c} (\mu ^{C(f),cal}_{y,\,lsg} - t) \}_{t \ge 1}$ is the vector of the values of the climate variable *c* in year *y* prior to the mean appearance time of leaf *f* on genotype *lsg*, and $\varphi (\cdot | \gamma )$ is a parametric function characterizing the impact of the longitudinal climate variables on the phyllochron (Additional file 1: Fig. S11A).

The line-year effect $\alpha _{y,\,l}$ accounts for the interaction between a genetic background and the conditions in a given year (climate and cultivation conditions including sowing date). The effect of each climate variable $c\in {\mathcal {C}}$ depends on the year-line background, which is accounted for by the coefficient $\beta _{y,\,l,\,j}$. Moreover, the year independent genotype effect is accounted for through the coefficient $\gamma _{lsc}$, and the residuals $\varepsilon _{y,\,lsg,\,f}$ are assumed to be i.i.d. (independent identically distributed) centered Gaussian variables.

#### Parameterization

The function $\varphi$ was parameterized as a piecewise constant function:

4 $$\begin{aligned} \varphi \left( \left\{ X_{y,\,c} \left(\mu ^{C(f),cal}_{y,\,lsg} - t\right) \right\}_{t \ge 1} | \gamma _{y,\,l,\,c} \right) = \sum _{w\in {\mathcal {W}}_0}\gamma _{y,\,l,\,c,\, w} \sum _t X_{y,\,c}\left(\mu ^{C(f),cal}_{y,\,lsg} -t\right) 1\!\text {I}_{t \le w } \end{aligned}$$

This flexible parameterization can model various phenomena with a small number of non-zero coefficients $(\gamma _{y,\,l,\,c,\, w})$, depending on the coefficient values (Additional file 1: Fig. S11B). We considered a set of time windows ${\mathcal {W}}_0=(1,2,3,5,10,20)$ days, to model both the effects over a short period of time and the potential long term impacts of the climate variables.

#### Inference with a lasso regression

The model was inferred by replacing unknown true phyllochron parameters (“$\mu$”) by their estimates computed by the Monte-Carlo EM algorithm (“${\widehat{\mu }}$”). To account for the large number of parameters in model (3) with parameterization (4), inference was performed using a lasso regression, which automatically sets to zero a large proportion of the coefficients by means of a $L^1$-penalty; the proportion of selected variables is tuned by a constant in the penalty. We used the R package penalized which allows to select the optimal penalty constant based on cross-validation. In order to preserve the structure of the data, cross-validation splitting was implemented by keeping together phyllochron measurements from each genotype-year combination.

Model performance was assessed by quantifying the prediction error. More precisely, for each genotype-year combination (*y*, *lsg*), the climate model (3, 4) was inferred from measurements of plants from all genotype-year combinations $(y', lsg') \ne (y, lsg)$. The predicted values of phyllochron parameters $(\mu _{y,\,lsg,\,f})_{f=f_{\min }^{y,\,lsg}+1, \ldots , f_{\max }^{y,\,lsg}}$ were then computed based on (3). Finally the Mean Squared Error (MSE) was computed between these predicted values and the values inferred with the model in the “A stochastic process model of the phyllochron” section, considered to be the “true” values.

#### Permutation-like test

The MSE may over-estimate the predictive ability of the model since the same climate data were used in the training and validation sets. To correct this bias, MSE calculations were repeated using “false” climates whose seasonal variations are not associated with phyllochron variation. False climate were generated using climate records from years 2009 to 2017 on three periods of time, leading to 252 false climates. Then, the proportion of false climates where the MSE was smaller than the one obtained with the “true” climate represents a *p*-value of the impact of climate on phyllochron, where the null hypothesis assumes no effect of climate on the phyllochron: $\gamma _{y,\,l,\,c,\,w}=0$ for all (*y*, *l*, *c*, *w*). Indeed, a low proportion indicates that the climate partly explains the phyllochron variations.

#### Weights

Lasso regression was implemented without and with weights accounting for the precision in the estimation of each $\mu _{y,\,lsg,\,f}$. More precisely, for each genotype *lsg* at year *y*, $\mu _{y,\,lsg,\,f}$ was weighted by the number of plants for which at least one observations was made before and after appearance of leaf *f*. Given the design of our dataset, the number of plants was smaller for higher leaf ranks as a portion of the plants have been dissected. Besides, as the number of plants depended on the year (smaller in 2014 and higher in 2016), the weights amounted to give unequal importance to the association between climate and phyllochron on the three years.

#### Which phyllochron estimates for the climate model?

Climate model was implemented using the phyllochron estimated from both the selected model (among parametric and complete models) and the complete model. While the selected model is expected to provide less noisy estimates than the complete model, parametric models create a dependence between successive $(\mu _f)_f$, which makes the interpretation more questionable. Indeed, consider e.g. the piecewise constant model with cut at leaf $\kappa$, then $\mu _{\kappa -2}$ and $\mu _{\kappa -1}$ are forced to be equal. Thus, as the value of the climate variables prior to the appearance of leaf of rank $\kappa -1$ are assumed to impact $\mu _{\kappa -1}$, they are also assumed to impact $\mu _{\kappa -2}$.

## Results

The results are gathered in three subsections. The “Analysis of the phyllochron model” subsection details the conceptual differences between successive time-to-event and regression models, based on simulated toy examples, and presents the model diagnosis. The “Statistical analysis” subsection gathers the main usage of the model: comparison between groups or conditions, and selection of the phyllochron dynamics or temporal trends. The last subsection is devoted to the climate model.

### Analysis of the phyllochron model

#### Successive time-to-event versus regression models: conceptual differences

**Fig. 3:**
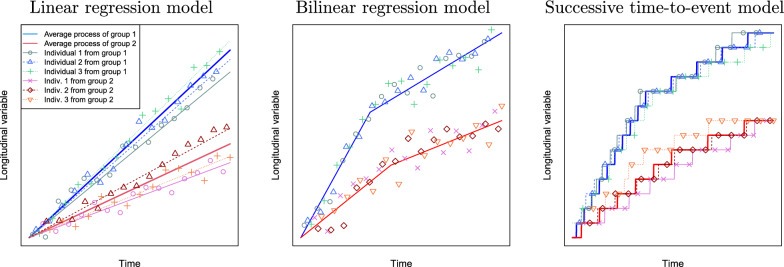
Illustration of regression and successive time-to-event models. Three types of modeling of a longitudinal count variable are shown: a linear regression model with individual effect (left), a bilinear regression model without individual effect (center) and a successive time-to-event model with two phases. For each model, observations were generated for six individuals belonging to two groups (points). Bold lines represent the average dynamics of the variable for each group, and thin lines the dynamics of each individual. For both regression models (linear with individual effect and non-linear without individual effect), the values generated at successive time points for a given individual are non-monotonous, unlike for the successive time-to-event model. In the context of phyllochron, the longitudinal variable corresponds to the number of visible leaves, the groups correspond to conditions or genotypes, and the individuals are plants. In the successive time-to-event model, segments (the duration between two jumps) of bold lines correspond to the parameters $(\mu _{y,\,lsg,\,f})_f$ while segments of thin lines correspond to the random variables $(Y_{y,\,lsg,\,p,\,f})_f$

The conceptual difference between regression and successive time-to-event modeling is illustrated in Fig. 3. Two regression models are shown: the classic linear phyllochron model with individual effect and an example of a non-linear model, the bilinear regression model without individual effect [15]. In both regression models, independent individual variations can take on negative or positive values, therefore the data generated for an individual (a plant in our context) are not increasing over time and cannot realistically model the number of leaves on a plant during growth. By contrast, with the time-to-event model, the values of successive observations of the longitudinal variable (the number of leaves) are always increasing.

The structure of the dependence between successive leaves can be analyzed at several levels: (i) the leaf number process, (ii) plant level variations within a genotype, (iii) the genotype level structure.

More precisely, at the level of the individual leaf number process, the assumptions of the regression model imply that measurements on the same plant are uncorrelated; by contrast, with the time-to-event model, a change in the phyllochron occurring at a certain leaf rank *f* (e.g. a shorter time $Y_f$ between the appearance of successive leaves) will impact the whole posterior trajectory because the number of leaves at time *t* results from the accumulated $(Y_f)_f$ of all the leaves *f* that have appeared until *t*. Therefore, if a given $Y_f$ is shorter than the genotype level mean, leaves after *f* will appear consistently later than the genotype level phyllochron dynamics.

Our model assumes independence of the plant level variations of the time between the appearance of successive leaves around the genotype level phyllochron values, thus the accelerating or decelerating trend of a plant phyllochron results from a deterministic process at the genotype level. Stochasticity in the random variables $Y_{y,\,lsg,\,f}$ comes from deviations from the average times between the appearance of successive leaves at the genotype level, captured by the residual variance parameter $\sigma_{y,lsg,f}^2$. These variations are likely to originate from a cumulation of independent causes, each with a small effect that can be either negative or positive (e.g. the quality of the light perceived by an individual plant during that time interval, the presence of insects, ...) irrespective of the time or the number of leaves already present.

Finally, unlike other models in the literature (linear or bilinear) our agnostic model does not assume any specific structure in the vector of genotype level mean time between the appearance of successive leaves in a genotype. Instead, parametric sub-models enable the exploration of various hypotheses regarding phyllochron structure, including classic temporal trends notably the linear leaf appearance process (constant $(\mu _{y,\,lsg,\,f})_f$) and the bilinear leaf appearance process (piecewise constant $(\mu _{y,\,lsg,\,f})_f$), while always generating an increasing leaf number process.

#### Model validation

**Fig. 4:**
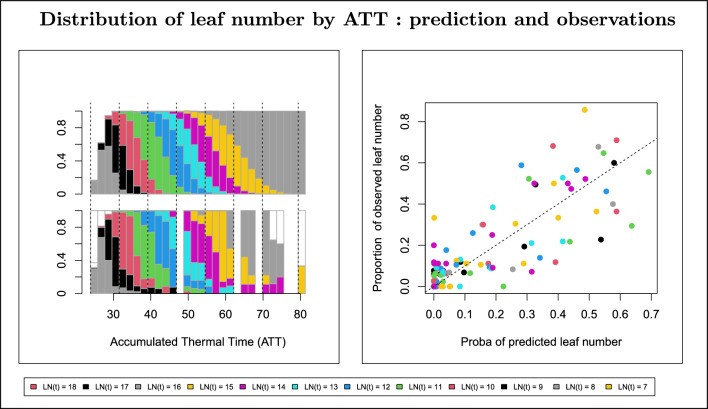
Predicted and observed leaf number distribution: model validation using genotype FL027 in year 2015 as an example. Left: the top graph shows the distribution of the number of visible leaves *LN*(*t*) predicted by the model at *t* within each ATT (Accumulated Thermal Time) interval. The bottom graph shows the distribution of the observed *LN*(*t*) measured in all plants of the genotype in that year within each ATT interval. Each color corresponds to a leaf rank within the range of modeled leaf ranks. White bars correspond to non-modelled leaf ranks. Vertical dashed lines were added to facilitate the visual comparison of the two graphs. Right: each point represents the proportion of observations of a given leaf rank within a given ATT interval as a function of its predicted counterpart. Leaf ranks are color coded; the dashed line is $y=x$

Interval censoring, where only an interval in which a leaf appears is known, means that the random variables $(Y_{y,\,lsg,\,p,\,f})_f$ are not observed and so it is not possible to check the fit of the model using the residuals $\mu _{y,\, lsg,\, f}- Y_{y,\, lsg,\, p,\,f}$. Nevertheless, the model can be indirectly validated by comparing the observed and predicted numbers of leaves at the same ATT (Fig. 4 and Additional file 1: S1). Indeed, for each genotype the model predicts the number of leaves on a plant at a given ATT as a random variable that can take on several values with an associated vector of probabilities. The probability plot allows a visual comparison of the theoretical (i.e. from the model) and empirical (i.e. from the observed data) probability distributions of the number of leaves at each time interval. The quantile-quantile plot shows more precisely the goodness of fit of the empirical and theoretical distributions. As an example, Fig. 4 shows the good fit between predictions and observations for genotype FL027 in year 2015. Note that the interpretation is different from classic quantile-quantile plots, specifically when checking the assumption of a normal distribution. Indeed, in the latter case empirical quantiles are compared with theoretical Gaussian quantiles with the same median, so by definition the plot is centered on the diagonal $y = x$ and is increasing, and the normality assumption is validated visually by the random distribution around the diagonal. By contrast, on the scatterplot of quantiles (Fig. 4), centering around the diagonal is indicative of goodness of fit. Indeed, if the mean length $\mu _{y,\,lsg,\,f}$ of the time interval during which the leaf number is equal to $(f-1)$ is under-estimated, then the predicted probability of observing $(f-1)$ leaves will tend to be under-estimated as well, so the points corresponding to leaf rank $(f-1)$ will tend to be below the diagonal.

### Statistical analysis of the estimated phyllochron

#### Comparison of the cumulated and instant phyllochron between genotypic groups

We conducted a hierarchical analysis of the effect of each grouping level (i.e. year, ancestral line, selection population and genotype) on the cumulated phyllochron, between sowing and the appearance of the first modelled leaf, and the instant phyllochron corresponding to the modelled leaf ranks. A detailed descriptive analysis is provided in Additional file 1: D and Fig. S3. In summary, the cumulated and the instant phyllochron were strongly impacted by year but in opposite direction (the faster the cumulated phyllochron, the slower the instant phyllochron). No general trend was found between selection population, but differences, preserved from one year to the next, were observed between genotypes from the same selection population.

Tests between genotypic groups were implemented only for years 2014 and 2015 when all genotypes were observed, and were restricted to the range of leaf ranks [8, 13] common to all genotypes. Table 4 summarizes the results of comparisons between ancestral lines, selection populations and genotypes with the permutation test. As a comparison, the *p*-values obtained with the simple $\chi ^2$-LRT (Additional file 1: Table S2) are globally smaller, which demonstrates the benefit of the permutation test procedure. Both cumulated and instant phyllochron were significantly different between genotypes derived from F252 and MBS ancestral lines. Differences between selection populations were only found in 2014, but may result from genotype effect, as underlined in the “Tests of genotypic groups effects” section. Permutation test preserving genotypes would enable to analyse selection effect out of genotype effect, but the small number of genotypes by selection population leads to a too limited number of permutations. Nevertheless, the graphical analysis based on PCA (Additional file 1: Fig. S9) shows no clear discrimination between Early and Late genotypes within each ancestral line. Finally, when the number of rows by genotype enables to generate a sufficient number of permutations, most genotypes within selection lines displayed a *p*-value smaller than 0.05 or between 0.05 and 0.07. The exception is MBS-late in 2015, for which the joint effect on cumulated and instant phyllochron was not significant ($p=0.37$), while the effect on the cumulated phyllochron only was significant ($p=0.029$). A potential explanation for this surprising result is the limited number of possible permutations (35) which makes the *p*-values less reliable.

The results of the statistical tests showing significant differences in global instant phyllochron between genotypic groups were complemented by the analysis at the level of the leaf rank of the temporal trends of the instant phyllochron dynamics.

#### Temporal trends in instant phyllochron

Additional file 1: Fig. S4 shows the estimates of the genotype level phyllochron, for all years and genotypes. While some temporal trends extend on several successive leaf ranks, phyllochron dynamics also exhibit less structured variations. The method for selecting the best sub-model among the parametric models and the complete model, which combines a statistical test and the AIC, enabled us to distinguish significant temporal trends from noise i.e. sampling variations. A complementary analysis at the row level was performed to ensure that temporal trends did not result from row effect (Additional file 1: D).

**Fig. 5:**
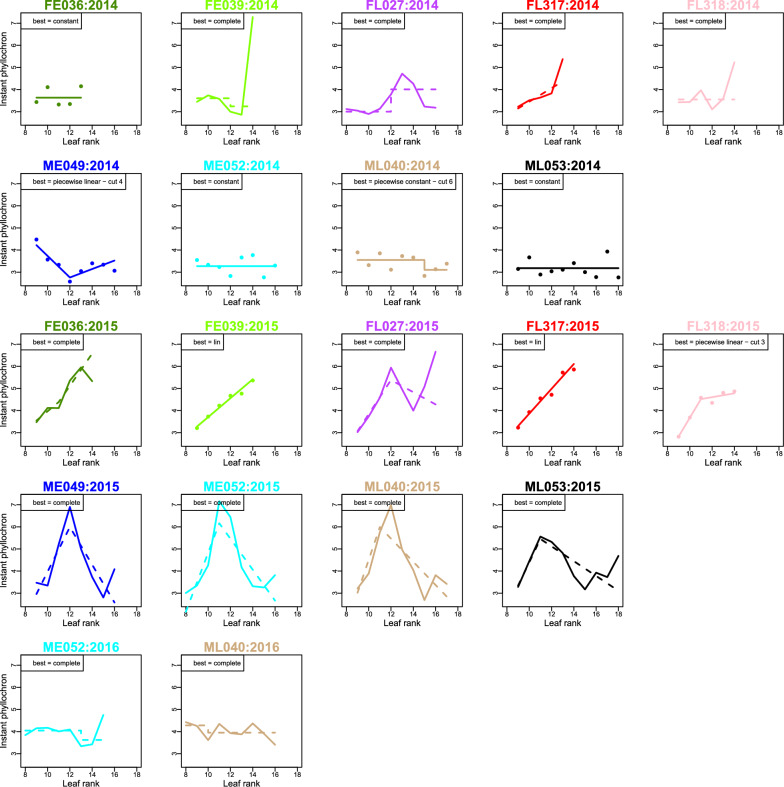
Best parametric sub-models. Each plot shows the instant phyllochron $({\widehat{\mu }}_{y,\,lsg,\,f})_{f=f_{\min }^{y,\,lsg} +1, \ldots ,f_{\max }^{y,\,lsg}}$ in degree-days for each genotype and each year. Solid line corresponds to the best model among all parametric sub-models and the complete model. When the best model corresponds to the complete model, the best parametric model was displayed in dashed line; when the best model is a parametric model, the complete model was plotted in dots. The best model is indicated on each plot

Additional file 1: Table S1 provides the AIC and $\chi ^2$-LRT *p*-values. The constant phyllochron model was predominantly rejected (for six of the nine genotypes in 2014 and for all genotypes in 2015 and 2016) and the complete model was selected in more than half of the genotype-year combinations. Figure 5 shows the model selected among the parametric models and the complete model as well as the best parametric model (if different), for each genotype-year combination. The selected model enlightens the temporal trends considered as significant by the selection procedure, while the best parametric model exhibits a relevant parsimonious representation which facilitates the comparison with existing models and the biological analysis. The most striking observation was the strong variations of temporal trends between years: in 2014 and 2016, the time between the appearance of successive leaves varied moderately along leaf ranks (1–2 degree days), except between leaves 13 and 14 for FE039 in 2014, while the variations were more important in 2015 (3–5 degree days). Differences between ancestral lines were mostly visible in year 2015: the ATT between successive leaves displayed a strong increasing phase followed by a decreasing phase in MBS genotypes with a maximum around leaves 11–12, while it increased in three genotypes of F252 (FL317, FE039, FL318), mostly increased in FE036 with a slight decrease at the last leaf rank and was more erratic for FL207. In 2014, the tendency to increase in the F252 genotypes was less clear, and the instant phyllochron of MBS genotypes appeared to be constant or decreasing. The two MBS genotypes in 2016 displayed a slightly decreasing trend.

### Input from climate variables

**Fig. 6:**
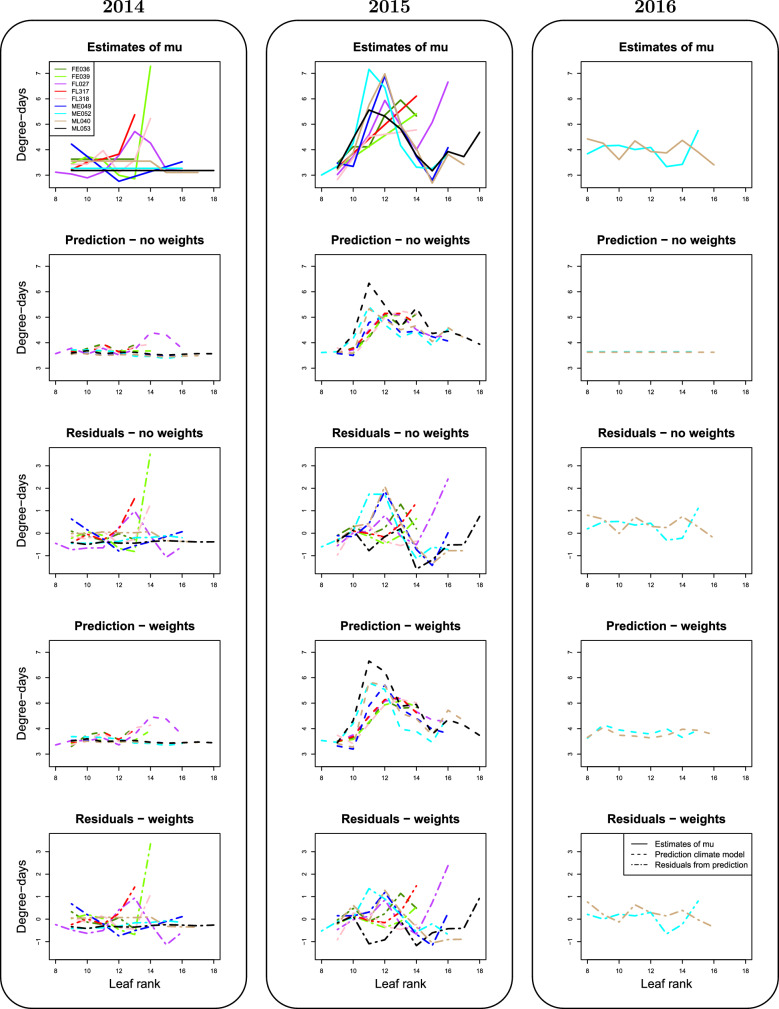
Predicted values and residuals of the climate model. The climate model is implemented using the phyllochron estimates with the best model selected among the parametric and complete models. Each plot corresponds to a year and each color to a genotype. Estimates of $(\mu _{y,\,lsg,\,f})$ as a function of leaf rank are shown in row 1; Rows 2 and 4 show the predicted values of ${\widehat{\mu }}_{y,\,lsg,\,f}$ with the climate model (3 and 4) in a cross-validation framework, for the unweighted and weighted procedure respectively. Rows 3 and 5 shows the residuals of the prediction i.e. the difference between estimate and prediction curves

Figure 6 and Additional file 1: Fig. S8 compare the estimates of $(\mu _{y, \,lsg,\,f})_{y,\,lsg,\,f}$ from the selected and the complete models respectively with the values predicted by the climate model (3, 4) in a cross-validation framework, both with and without weights accounting for the number of observations used to compute each $\mu _{y,\,lsg,\, f}$.

The top row provides the phyllochron estimates for each year separately. Rows 2 and 4 give the predictions from the climate model with and without weights. Rows 3 and 5 show the temporal fluctuations of the residuals of the climate model. Correspondence between temporal trends in the estimates and predictions from the climate model asseses in which extent seasonal climate fluctuations are able to explain phyllochron variations. According to the climate model, residuals are supposed to be independent of the predicted values and should not exhibit any temporal pattern. Thus, the significant temporal trends observed in 2015 were partially recovered by the climate model. This is confirmed by the much smaller range of phyllochron variations in the residuals (${-}$ 1/${+}$ 1.5 degree-days) than in the phyllochron estimates (3–7 degree-days). Nevertheless, weaker variations were not captured by the model; for example, in 2016 the predicted phyllochrons are almost constant for the model without weights, and capture a weak part of the variations for the model with weights, and observed temporal variation can be found in the temporal patterns of the residuals. Besides, the weighted climatic model leads to an improvment of the cross-validation predictions for the 2016 genotypes and a degradation for the genotypes from 2014, with respect to the unweighted model. As weighting gives more importance to the phyllochron from 2016 in the model inferrence (due to a larger number of observed plants), this suggests that prediction is improved when inferrence is performed on genotypes from the same year and that the association between climate and phyllochron could be modulated from one year to the other.

As highlighted in the “Permutation-like test” section, despite cross-validation, the predictions from the climate model are not totally independent since the same climate records were used in the training and the validation sets. Therefore, cross-validation predictions were computed with 252 “false” climates (see the “Permutation-like test” section), and the Mean Squared Error (MSE) was defined as:

5 $$\begin{aligned} MSE = \sum _{y,\,lsg,\,f} \left( \mu ^{pred}_{y,\,lsg,\, f} - {\widehat{\mu }}_{y,\,lsg,\,f} \right) ^2\omega _{y,\,lsg,\,f} \end{aligned}$$

with $(\mu ^{pred}_{y,\,lsg,\, f})$ the phyllochrons predicted with the climate model (3, 4) converted to thermal time, and $(\omega _{y,\,lsg,\,f})$ the weights, equal to 1 for the unweighted climate model. The smaller the MSE, the better prediction. Results in Table 5 indicate a significant effect of the climate on the phyllochron when weights accounting for the uncertainty in the estimation of the $(\mu _{y,\,lsg,\,f})$ are used ($p= 0.052$ for the best phyllochron model and 0.032 for the complete phyllochron model). Without weights, the effect appears weaker ($p= 0.123$ for the best phyllochron model and 0.044 for the complete phyllochron model). With and without weights, the MSE is smaller when the complete phyllochron model is considered. An hypothesis is that some variations which were not selected by the model selection procedure could be biologically relevant. Another possible explanation is related to the correlation between the estimates of successive $(\mu _{u,lsg,f})_f$ induced by the parametric models, as detailed in the “Materials and methods” section.

Lasso regression is often used to select features by keeping variables with non-zero coefficients. However, here, this selection method is not robust as the climate variables are strongly correlated (Additional file 1: Fig. S12). Therefore, we chose not to display a list that could lead to misleading interpretations. But since phyllochrons exhibited a particular structure in 2015, we proposed a descriptive analysis of some pre-specified climatic variable (see Additional file 1: D).

## Discussion

### A stochastic process model of the phyllochron

In this paper, we propose a hypothesis testing model of the phyllochron based on a successive time-to-event modeling where the phyllochron varies non-parametrically with leaf rank, which addresses some flaws of the classic linear phyllochron model, mostly used for hypothesis testing [10]. First of all, the flexible structure of our model allows for more accurate modeling. Indeed, our results show strong variations in the phyllochron throughout a season, indicating that the *linear model*, which assumes constancy of the leaf appearance rate, may be too simple under particular climate conditions. Moreover, our analysis of various parametric sub-models suggests that a two-phase model could represent a reasonable compromise between simplicity and flexibility, even if the complete model is often statistically more accurate. Bilinear models have been proposed in the literature for phyllochron analysis [15] and for prediction models (e.g. APSIM, [35]), but they do not allow hypothesis testing.

Secondly, our model represents a more realistic modeling of plant level variations and is conceptually different from regression models, which ignore the correlation between leaf numbers at successive times on the same plant, and thus may generate bias in statistical tests. In addition, we propose an unbiased statistical procedure to compare phyllochrons under various conditions, which we applied to genotypic group comparisons. However, in its present version our algorithm can not estimate a model with covariates, and the comparison between groups or conditions is performed by a permutation test, which limits the complexity of the framework. On the one hand, framework complexity related to the effects of interest is restricted to two-by-two model comparison and cannot handle e.g. hierarchical effects. On the other hand, clusters generated by experimental conditions, usually modelled by random effects in mixed models, are accounted for by constraints on the permutations, which requires a sufficient number of clusters to get a minimum number of permutations and determines the precision of the *p*-value.

Besides, our model assumes independence between the plant level variations around the genotype mean phyllochron dynamics, thus the accelerating or decelerating trends in a plant phyllochron only result from a deterministic process at the genotype level. This assumption is a limitation of our current algorithm. Neverthless, neither dependence assumptions or independence at the plant-level model could be directly checked with interval censored data, and the comparison of the observations and the predictions based on our model at the same ATT shows a good concordance.

Generally, a price to pay for flexibility is having a larger number of parameters, which requires more data to be correctly estimated. This limitation is particularly true in the context of interval censoring, where information is restricted to time intervals during which leaves appear. Nevertheless, our results indicate that approximately 50 plants per genotype with complete phyllochron observations (in 2015) were sufficient to highlight significant differences between genotypic groups resulting from a recent divergence.

The impact of climate on the phyllochron variations through season was assessed using a two-step procedure: first, the parameters of the phyllochron model were inferred without taking climate into account, then the cumulative climate variables before the appearance of each leaf were regressed on the genotype level phyllochron. Two-step inference processes have been pointed out as potentially biased in other statistical contexts (e.g. in clustering [36]), notably as the variance of the parameter estimates is not accounted for. But we partially circumvented this bias by weighting each phyllochron parameter estimate by the number of “useful” observations for the estimation of this parameter and the use of a permutation test limits the potential bias. Moreover, a two-step inference remains a standard method when full-likelihood minimization is not available. Notably, it is classically used in genotype-to-phenotype studies where a dynamic phenotype is summarized by a restricted set of parameters on which statistical analyses are performed [37, 38]. It may be noted that since our climate model is based on the inferred genotype level phyllochron and does not make use of the plant level variation modeling, the impact of using a successive time-to-event model instead of a regression model is less crucial than for hypothesis testing, since these two classes of model mainly differs on the plant level variations around the genotype level phyllochron.

Our model assumes Gaussian plant level variations of the phyllochron around the genotype level phyllochron, which allowed us to use existing tools to sample from truncated multivariate distributions. Approximation of a positive variable by a Gaussian distribution is acceptable if the standard deviation is smaller than three to four times the mean, which is the case for most but not all the genotypes in our data set (Additional file 1: Fig. S14). Besides, Gaussian approximations are very commonly used in statistical modeling, and lead to unbiased or moderately biased results in diverse contexts. Moreover, in our model, the stochasticity in the random variables $Y_{y,\,lsg,\,f}$ comes from deviations from the mean genotype value of the time between the appearance of successive leaves. Such deviations are likely to originate from a cumulation of independent causes, each with a small effect that can be either negative or positive (e.g. quality of the light perceived by an individual plant during the interval, presence of insects, ...). In probability theory, the central limit theorem establishes that, in many situations, when independent random variables are summed up, their sum tends toward a normal distribution even if the original variables themselves are not normally distributed. More generally, in the context of interval censoring where leaf appearance time is not observed, assumptions regarding the distribution of the time lengths between the appearance of successive leaves cannot be directly checked by the analysis of the residuals. Nevertheless, we carried out validations by comparing the observed values and the model predictions, showing the validity of the reconstruction.

This work is in line with recent approaches for a unique time-to-event analysis in agriculture [21, 22] that were developed as an alternative to flawed regression models, even though phyllochron modeling, which includes successive events, is more cumbersome. However, regression models remains much more widely used, probably due to their simpler formulation and much lower computing complexity. Therefore, the next step would be to quantitatively assess the potential bias of regression model and the benefits of a more accurate modelling, and evaluate in which situations it would be worth having to carry on in this direction. Regarding phyllochron, differences are not expected on the estimated temporal trend, but rather in the testing procedures, which make a direct use of the distribution of the plant level variations. Thus, further works could target the impact of each factor (number of plant by conditions, number of time-point by year, non-constancy of leaf appearance rate accross the season, etc) on the performance of successive time-to-event and regression-based phyllochron models.

Moreover, we are currently working on a more general framework based on semi-Markov models that could directly incorporate longitudinal covariates such as climate variables into the phyllochron model. This class of stochastic process can also handle more general distributions and thus relax the normality assumption. Our first results indicate that the assumption of Gaussian plant level variations has practically no impact on the genotype level phyllochron estimates. Semi-Markov models are a growing topic with diverse application domains, and future statistical and algorithm work could allow the inclusion of more complex covariates, and address, within a more accurate statistical framework, complex designs that are currently tested with the constant phyllochron model.

### Structure of phyllochron

A unique feature of our model is that it does not assume any structure in the mean time between the appearance of successive leaves for a given genotype and year. This is quite novel compared to regression models, which assume a constant time between appearance of successive leaves over a season, or bilinear regression models, which only allow two different values. In FSPMs, the structure is imposed by strong biological hypotheses that are still debated in the community. For example, assumptions of a constant plastochron (leaf production rate) and a variable leaf elongation rate are common (e.g. [39]). However, phenotypes at the scale of the whole plant (e.g. leaf appearance) result from multiple complex mechanisms at the level of plant organs that are difficult to observe. In the FSPM model, allowing for variation over time of the plastochron would result in a different estimate of elongation rates or of their dependency to environmental factors and very different models could equally fit observed data at the plant level. In our model, we do not make any biological hypotheses regarding the underlying mechanisms.

Indeed, we observed wide fluctuations in the genotype level phyllochron between successive leaves, at least in the year 2015. This result may appear surprising with regard to the literature, but these estimates mostly result from strong modeling hypotheses. For example, in [20] although a constant leaf appearance rate was assumed and inferred by linear regression, the raw data (Figure 1b) clearly displayed a temporal trend around the regression line.

Our agnostic model enables us to explore the hypothesis of a structured phyllochron through the comparison of parametric sub-models, which include the classic temporal trends considered in literature, notably the linear and bilinear leaf appearance process, but with a more realistic model of the leaf appearance process at the plant level.

### Differences in phyllochron between closely related genotypes

Due to experimental constraints, for global comparisons, we modeled the phyllochron on a restricted range of leaf ranks (8–13). Additionally, we used the cumulated time between sowing and the appearance of leaf 8 as an indicator of the first growing phase of plant development, i.e. from seedling emergence to the first phase of the phyllochron.

As previously described, maize lines produced by Saclay’s Divergent Selection Experiments (DSEs) exhibit a gradual flowering time divergence over the first 13 generations [23, 24]. Characterizing the phyllochron of these genotypes, as performed here, helps to better understand the developmental changes that could underlie this response to selection. Our analyses did not highlight differences in the phyllochron of selection populations (beyond genotype-level effects). Nevertheless, we observed that the total number of leaves was impacted during the selection process, with Late genotypes tending to produce more leaves than Early genotypes (Additional file 1: D) and a change in leaf number is sufficient to accelerate or delay the flowering time [40]. However, the ability to discriminate between genotype and selection population effects may be limited by the small number of genotypes per selection population considered.

Differences in phyllochron have previously been reported between genetically distant maize inbred lines [41] and between hybrids [10]. Interestingly, our results indicate phyllochron differences between closely related genotypes and notably between genotypes selected in response to the same selective pressure (early or late flowering), suggesting that the phyllochron evolved independently of the selection pressure for flowering time. These differences appear robust to environmental variations observed from one year to the next. They could be explained by random mutations appearing during the selection process, which may or may not contribute to the selection response.

### Temporal trends in phyllochron dynamics and climate

The phyllochron was found to be significantly non-constant for a majority of genotype-year combinations. Importantly, the model does not favor any temporal trend, thus the observed phyllochron dynamics only originated from the data. Notably, we found strong seasonal variations in the phyllochron in 2015, a year with particular climate conditions, and this dynamic trend was associated with the ancestral line, i.e. MBS genotypes displayed an increasing and a decreasing phase whereas F252 genotypes displayed two increasing phases. This suggests that there are interactions between the environment and ancestral lines, as previously reported in [42]. However, since different genotypes do not experience the same environmental conditions at the same developmental stage, these differences may originate either from selection or from the environmental conditions [13]. Therefore, we proposed a model that includes longitudinal climate variables and thus enables a calendar time analysis, and assumes ancestral line-climate interactions.

Our results indicate that taken together, the climate variables partly explain the strong seasonal variations in phyllochron observed in 2015, but do not explain the more moderate variations. The strong correlations between climate variables, which are for the most part preserved from one year to the next, would make variable selection non-robust and the interpretation of the selected features potentially misleading. Qualitatively, year 2015 was particularly dry and sunny from the beginning of May to mid-July, which could explain the general increase in leaf appearance time over this period. The two-phase phyllochron observed in 2015 questions the idea that the phyllochron is constant. This pattern may arise from a two-phase response to drought: a slowing down of leaf appearance allowing the plant to tolerate short term drought, followed by a recovery of the initial rate allowing the plant to complete its life cycle if drought conditions persist. Indeed, [43] induced such a response in maize using shelters to prevent rainfall for three weeks in 10-leaved plants. As mentioned above, the classic assumption of a constant phyllochron may prevent the precise detection of this type of pattern. Furthermore, other physiological observations in maize during drought stress such as changes in leaf elongation rates, photosynthesis and transpiration rates as well as leaf rolling could result in changes in leaf appearance rates [44, 45]. The effect of irradiance on the phyllochron has been previously investigated leading to different conclusions depending on the genotype. While [26, 27] showed that decreased irradiance slows down leaf emergence, [16] detected the opposite effect of irradiance on leaf emergence rate.

Irrespective of the limitations of the two-step approach discussed above, additive modeling of covariate effects may not be enough to recover the complex interplay between climate variables. Notably, the model does not account for a potential non-monotonous effect of the variables (medium optimum value). Nevertheless, the price to pay for a richer model would be a large number of parameters with respect to the number of phyllochron estimates used for model fitting, and such a model could not be properly inferred with our limited number of genotype-year combinations. Indeed, one study investigating the non-additive effects of drought and temperature on crop yield required more than 50 years of climate variables [46]

### Conclusion

We developed a hypothesis-testing model of phyllochron dynamics based on a successive time-to-event stochastic process, combining a flexible and accurate modeling with an unbiased statistical testing procedure. The model detected fine-scale differences between related genotypes with a moderate experimental effort (10–20 measurements throughout the season on 30–50 plants per genotype). The comparison of our model with the classic regression models of phyllochron in terms of statistical testing would quantitatively assess the benefit of this more accurate modelling. On the DSE dataset, we showed that the major sources of differences in phyllochron were not the selection population (Early or Late), but rather the ancestral line (F252 or MBS), the year of experimentation, and the leaf rank. Moreover, our results clearly indicate that the phyllochron is not always constant throughout the season, and these temporal trends could be associated with climate. These biological findings could be validated by applying the phyllochron model to broader data-sets [20].

**Table 1:** Hierarchical structure of the plant material

Ancestral line	Selection population	Genotype
F252	Early	FE036, FE039
F252	Late	FL027, FL317, FL318
MBS	Early	ME049, ME052
MBS	Late	ML040, ML053

**Table 2:** Climate variables collected from the INRAE database Climatik [31]

Climate variable	Unit	Abbreviations
Temperature range	$^\circ$C	TR
Penman evapotranspiration	mm	PETP
Calculated global radiation	$\hbox {J cm}^{-2}$	CGR
Global radiation	$\hbox {J cm}^{-2}$	GR
Photosynthetically active radiation	$\hbox {J cm}^{-2}$	PAR
Calculated sunshine duration	h	CID
Pourcentage of sunshine	%	IF
Rain falls	mm	RF
Maximum rain falls	mm h$^{-1}$	RFX
> 40% humidity duration	h	HD4
Wetness duration	h	WD
Maximum humidity	%	HX
> 90% humidity duration	h	HD9
Minimum humidity	%	HN
> 80% humidity duration	h	HD8
Atmospheric humidity	%	H
Wind speed	m s$^{-1}$	W
Maximum wind speed	m s$^{-1}$	WX
Dewpoint temperature	$^\circ$C	DT
Mean vapor pressure	mbar	MVP
Actinothermic index 10 cm	$^\circ$C	AI1
Actinothermic index 50 cm	$^\circ$C	AI5
Minimum temperature	$^\circ$C	TN
Mean temperature	$^\circ$C	T
Calculated mean temperature	$^\circ$C	CMT
Maximum temperature	$^\circ$C	TX
Maximum actinothermic index 10 cm	$^\circ$C	I1X
Maximum actinothermic index 50 cm	$^\circ$C	I5X

**Table 3:** Summary of notations

Notation	Description
*y*	Index for year
*p*	Index for plant
*lsg*	Index for the $g{\textrm{th}}$ genotype in selection population *s* from inbred line *l*
*f*	Index for leaf rank
$[f_{\min }^{y,\,lsg}, f_{\max }^{y,\,lsg}]$	Largest interval of leaf ranks such that leaves $f_{\min }^{y,\,lsg}-1$ and $f_{\max }^{y,\,lsg}$ are observed on at least 10 plants from genotype *lsg* on year *y*
$[f_{\min }^0, f_{\max }^0]$	Common interval to all genotypes and years, equal to $\bigcap \limits _{y,\,lsg}[f_{\min }^{y,\,lsg}, f_{\max }^{y,\,lsg}]$
$F_{ lsg}$	Overall maximum number of leaves for genotype *lsg*
$H_{y,\,lsg,\,p,\,f}$	Time between sowing and appearance of leaf *f* on plant *p* from genotype *lsg* on year *y* (in degree-days)
$Y_{y,\,lsg,\,p,\,f}$	Time between appearance of leaf $f-1$ and *f* on plant *p* from genotype *lsg* on year *y* (in degree-days)
$\mu _{y,\,lsg,\,f}$	Mean of $Y_{y,\,lsg,\,p,\,f}$ i.e. mean interval between appearance of leaf $f-1$ and *f* for genotype *lsg* on year *y* (in degree-days)
$\mu _{y,\,lsg}^{C\left(f_{\min }\right)}$	Mean of $H_{y,\,lsg,\,p,\,f_{\min }}$ i.e. mean time between sowing and appearance of leaf $f_{\min }$ for genotype *lsg* on year *y* (in degree-days)
$\sigma _{y,\,lsg,\,f}$	Standard deviation of $Y_{y,\,lsg,\,p,\,f}$ (in degree-days)
$\sigma ^{C(f_{\min })}_{y,\,lsg}$	Standard deviation of $H_{y,\,lsg,\,p,\,f_{\min }}$ (in degree-days)
$X_{y,c}(t)$	Value of the climatic variable *c* on day *t* from year *y*
${\overline{\mu }}^{cal}_{y,\,lsg,\,f}$	Mean calendar time between sowing and appearance of leaf *f* for genotype *lsg* on year *y* (in days)
Plant level phyllochron	Vector $(Y_{y,\,lsg,\,p,\,f})_{f=1, \ldots , F_{lsg}}$ of times between appearance of successive leaves.
Genotype level phyllochron	Vector $(\mu _{y,\,lsg,\,f})_{f=1, \ldots , F_{lsg}}$ of mean times between appearance of successive leaves over the genotype (*y*, *lsg*)

**Table 4:** Models and *p*-values of the permutation test based on LRT statistics

Model	Cumulated phyllochron parameters	Instant phyllochron parameters	Model name		
(A) Models for genotypic groups effects (C: cumulated phyllochron, I: instant phyllochron)					
$M_{00}$	$\forall (l,s,g), \quad (\mu ^{C(f_{\text{min}}^0)}_{y,lsg},\sigma ^{C(f_{\text{min}}^0)}_ {y,lsg}) = (\mu ^{C(f_{\text{min}}^0)}_y,\sigma ^{C(f_{\text{min}}^0)}_y)$	$\forall (l,s,g), \quad (\mu _{y,lsg},\sigma _ {y,lsg}) = (\mu_y , \sigma_y )$	($\hbox {C}+\hbox {I}$)[identical]		
$M_{10}$	$\forall (s,g), \quad (\mu ^{C(f_{\text{min}}^0)}_{y,lsg},\sigma ^{C(f_{\text{min}}^0)}_ {y,lsg}) = (\mu ^{C(f_{\text{min}}^0)}_{y,l} ,\sigma ^{C(f_{\text{min}}^0)}_{y,l})$	$\forall (l, s,g), \quad (\mu _{y,lsg},\sigma _ {y,lsg}) = (\mu_y ,\sigma_y )$	C[line]-I[identical]		
$M_{11}$	$\forall (s,g), \quad (\mu ^{C(f_{\text{min}}^0)}_{y,lsg},\sigma ^{C(f_{\text{min}}^0)}_ {y,lsg}) = (\mu ^{C(f_{\text{min}}^0)}_{y,l},\sigma ^{C(f_{\text{min}}^0)}_ {y,l})$	$\forall (s,g), \quad (\mu _{y,lsg},\sigma _ {y,lsg}) =(\mu _{y,l},\sigma _ {y,l})$	($\hbox {C}+\hbox {I}$)[line]		
$M_{21}$	$\forall g, \quad (\mu ^{C(f_{\text{min}}^0)}_{y,lsg},\sigma ^{C(f_{\text{min}}^0)}_ {y,lsg}) = (\mu ^{C(f_{\text{min}}^0)}_{y,ls},\sigma ^{C(f_{\text{min}}^0)}_ {y,ls})$	$\forall (s,g), \quad (\mu _{y,lsg},\sigma _ {y,lsg})=(\mu _{y,l},\sigma _ {y,l})$	C[selection]-I[line]		
$M_{22}$	$\forall g, \quad (\mu ^{C(f_{\text{min}}^0)}_{y,lsg},\sigma ^{C(f_{\text{min}}^0)}_ {y,lsg}) = (\mu ^{C(f_{\text{min}}^0)}_{y,ls},\sigma ^{C(f_{\text{min}}^0)}_ {y,ls})$	$\forall g, \quad (\mu _{y,lsg},\sigma _ {y,lsg}) =(\mu _{y,ls},\sigma _ {y,ls})$	($\hbox {C}+\hbox {I}$)[selection]		
$M_{32}$	$\quad (\mu ^{C(f_{\text{min}}^0)}_{y,lsg},\sigma ^{C(f_{\text{min}}^0)}_ {y,lsg})= (\mu ^{C(f_{\text{min}}^0)}_{y,lsg},\sigma ^{C(f_{\text{min}}^0)}_ {y,lsg})$	$\forall g, \quad (\mu _{y,lsg},\sigma _ {y,lsg}) = (\mu _{y,ls},\sigma _ {y,ls})$	C[genotype]-I[selection]		
$M_{33}$	$(\mu ^{C(f_{\text{min}}^0)}_{y,lsg},\sigma ^{C(f_{\text{min}}^0)}_ {y,lsg}) = (\mu ^{C(f_{\text{min}}^0)}_{y,lsg},\sigma ^{C(f_{\text{min}}^0)}_ {y,lsg})$	$(\mu _{y,lsg},\sigma _ {y,lsg}) = (\mu _{y,lsg},\sigma _ {y,lsg})$	($\hbox {C}+\hbox {I}$)[genotype]		

	$M_{11}$/$M_{00}$	$M_{11}$/$M_{10}$	$M_{10}$/$M_{00}$	Nb of perm	Best
	Line effect on ($\hbox {C}+\hbox {I}$)	Line effect on I	Line effect on C	Nb of perm	
(B) Ancestral line effect					
2014	0.000	0.0100	0.01	200	$\hbox {C}+\hbox {I}$
2015	0.000	0.0000	0.0050	200	$\hbox {C}+\hbox {I}$

	$M_{22}$/$M_{11}$	$M_{22}$/$M_{21}$	$M_{21}$/$M_{11}$	Nb of perm	Best
	Selection effect on ($\hbox {C}+\hbox {I}$)	Selection effect on I	Selection effect on C	Nb of perm	
(C) Selection effect					
2014.F	0.043	0.0095	0.49	200	I
2014.M	0.540	0.4900	0.49	70	ns
2015.F	0.210	0.1400	1.0000	200	ns
2015.M	0.380	0.6700	0.1800	200	ns

	$M_{33}$/$M_{22}$	$M_{33}$/$M_{32}$	$M_{32}$/$M_{22}$	Nb of perm	Best
	Genotype effect on ($\hbox {C}+\hbox {I}$)	Genotype effect on I	Genotype effect on C	Nb of perm	
(D) Residual genotypic effect					
2014.Fearly	NA	NA	NA	6	NA
2014.Flate	0.067	0.0000	0.47	90	I
2014.Mearly	NA	NA	NA	6	NA
2014.Mlate	NA	NA	NA	6	NA
2015.Fearly	0.056	0.0079	1.0000	200	I
2015.Flate	0.025	0.0250	0.0700	200	I
2015.Mearly	0.000	0.0160	0.0079	200	$\hbox {C}+\hbox {I}$
2015.Mlate	0.370	0.4600	0.0290	35	C

(A) Definition of the models; (B–D) *p*-values for ancestral line, selection and genotypic effects. For each grouping effect, models $M_{j-1,j-1}$, $M_{j-1,j}$, and $M_{j,j}$ were compared. The best model shows either no group effect (ns), a significant group effect on the cumulative phyllochron (C), a significant group effect on the instant phyllochron (I) or both $(\hbox {C}+\hbox {I})$, at level $p<0.05$. Column “nb of perm” provides the number of permutation used to compute the empirical distribution under the parsimonious model

**Table 5:** Proportion of the false climates for wich the MSE (5) was smaller than the one obtained with the true climate model, when the climate model is computed either with and without weighting by the number of plants such that the time of appearance of leaf *f* is lower and upper bounded

	No weights	Weights
Best model for $\mu$	0.123	0.052
Complete model for $\mu$	0.044	0.032

## Supplementary Information

**Additional file 1.** Provides technical details regarding mathematical methods and statistical analyses, together with secondary results and figures.

## Data Availability

All scripts and datasets supporting the this article are available at the INRAE data respository [https://doi.org/10.15454/CUEHO6].

## Abbreviations

ATT: Accumulated thermal time
DSE: Divergent selection experiment
PCA: Principal component analysis
MSE: Mean squared error
LRT: Likelihood ratio test
