# Supplementary material for: A successive time-to-event model of phyllochron dynamics for hypothesis testing: application to the analysis of genetic and environmental effects in maize
Source: Plant Methods. 2023 Jun 7;19:54. doi: 10.1186/s13007-023-01029-7 (PMC10245529; doi:10.1186/s13007-023-01029-7)
Supplement: Supplementary file 1 — Additional file 1. Provides technical details regarding mathematical methods and statistical analyses, together with secondary results and figures. [file 13007_2023_1029_MOESM1_ESM.pdf]

Supplementary Material

A successive time-to-event model of phyllochron dynamics for hypothesis testing - application to the analysis of genetic and environment effects in maize

S. Plancade, E. Marchadier, S. Huet, A. Ressayre, C. Noûs, C. Dillmann

A Monte Carlo Expectation Maximization Algorithm

In this section, we consider a given genotype lsg (or higher level genotypic group) and a given year y, so the indexes lsg and y are omitted. For a plant p, let $(t_{p,1}, \dots, t_{p,N_p})$ be the monitoring time points (with sowing at $t = 0$) and $X_{p,j}$ the number of leaves of plant p at time $t_{p,j}$. For each plant and leaf rank, the observations indicate that leaf f appeared some time between the last observation date when the plant had at most $(f-1)$ leaves, and the first observation date when the plant had at least f leaves. We denote by $\nu_{p,f}$ and $\tau_{p,f}$ these two dates, respectively:

$$\nu_{p,f} = \begin{cases} \max\{t_{p,j}, j \text{ s.t. } X_{p,j} \leq f\} & \text{if } \min(X_{p,j}) \leq f \\ 0 & \text{otherwise} \end{cases}$$

$$\tau_{p,f} = \begin{cases} \min\{t_{p,j}, j \text{ s.t. } X_{p,j} > f\} & \text{if } \max(X_{p,j}) > f \\ +\infty & \text{otherwise} \end{cases}$$

The likelihood of the observations involves an integral of dimension $f_{\max} - f_{\min} + 1$, thus direct maximization is intractable. Consequently, we used a Monte-Carlo Expectation Maximization algorithm, where the *complete data* (unobserved) are $\mathbf{H}_p = (H_{p,f})_{p=1,\dots,n, f=f_{\min},\dots,f_{\max}}$

- Initialization. Proxies of $\{H_{p,f}\}_{p,f}$ are computed as:

$$\widehat{H_{p,f}} = \frac{1}{2}(\nu_{p,f} + \tau_{p,f}), \quad f = f_{\min}, \dots, f_{\max}$$

  for all (p, f) such that $\nu_{p,f} > 0$ and $\tau_{p,f} < \infty$, and the initial values of μ_f and σ_f are inferred as robust estimators of proxy-based mean and variance.

- Monte-Carlo E-step. Let $\Theta^{(m)} = (\mu^{C,(m)}, \sigma^{C,(m)}, \mu_f^{(m)}, \sigma_f^{(m)})_{f=f_{\min}+1,\dots,f_{\max}}$ be the current value of the parameters. For each p a sample $(\mathbf{H}_{p,r}^{(m)})_{r=1,\dots,N}$ is generated from the conditional distribution:

$$\mathbb{P}_{\Theta^{(m)}}[\mathbf{H}_p | H_{p,f} \in [\nu_{p,f} + \tau_{p,f}), f = f_{\min}, \dots, f_{\max}]$$

  namely a truncated multivariate Gaussian distribution. The computation time of classic rejection methods dramatically s with dimension, however specific tools are available for multivariate normal distributions. We used the R package `TruncatedNormal` (Botev and Belzile, 2020), which is well adapted when the truncated region is in the tail of the distribution.

- M-step. For $f = f_{\min} + 1, \dots, f_{\max}$, let $Y_{p,r,f}^{(m)} = H_{p,r,f}^{(m)} - Y_{p,r,f-1}^{(m)}$. The new value of the parameters $\Theta^{(m+1)}$ maximizes

$$\sum_{f=f_{\min}+1}^{f_{\max}} \left(\sum_{p=1}^{n} \sum_{r=1}^{N} \log \phi_{\mu_f, \sigma_f}(Y_{p,r,f}^{(m)}) \right) + \sum_{p=1}^{n} \sum_{r=1}^{N} \log \phi_{\mu_{f_{\min}}^C, \sigma_{f_{\min}}^C}(H_{p,r,f_{\min}}^{(m)}) \quad (1)$$

  with $\phi_{\mu,\sigma}$ the density of the normal distribution of mean μ and standard deviation σ. This problem is equivalent to a simple maximum likelihood estimation of a multivariate Gaussian distribution with a diagonal covariance matrix, thus:

$$\mu_f^{(m+1)} = \frac{1}{nN}\sum_{p=1}^{n}\sum_{r=1}^{N} Y_{p,r,f}^{(m)}, \quad f = f_{\min}+1,\ldots,f_{\max} \tag{2}$$
$$\mu^{C,(m+1)} = \frac{1}{nN}\sum_{p=1}^{n}\sum_{r=1}^{N} H_{p,r,f_{\min}}^{(m)}$$
$$\left(\sigma_f^{(m+1)}\right)^2 = \frac{1}{nN}\sum_{p=1}^{n}\sum_{r=1}^{N}\left(Y_{p,r,f}^{(m)}\right)^2 - \left(\mu_f^{(m+1)}\right)^2, \quad f = f_{\min}+1,\ldots,f_{\max}$$
$$\left(\sigma^{C,(m+1)}\right)^2 = \frac{1}{nN}\sum_{p=1}^{n}\sum_{r=1}^{N}\left(H_{p,r,f_{\min}}^{(m)}\right)^2 - \left(\mu^{C,(m+1)}\right)^2$$

The algorithm is iterated until stabilization of the parameters.

B Parametric sub-models

For each genotype lsg and year y, we considered the following parametric models for the average interval times between leaves $\mu_{y,lsg,f_{\min}^{lsg}+1},\ldots,\mu_{y,lsg,f_{\max}^{y,lsg}}$ as a function of leaf rank:

- Constant: $\mu_{y,lsg,f} = a_{y,lsg}$, $\forall f = f_{\min}^{y,lsg}+1,\ldots,f_{\max}^{y,lsg}$ (mod-ct)
- Linear: $\mu_{y,lsg,f} = a_{y,lsg} \times f + b_{y,lsg}$, $\forall f = f_{\min}^{y,lsg}+1,\ldots,f_{\max}^{y,lsg}$ (mod-lin)
- Piecewise constant: $\mu_{y,lsg,f} = a_{lsg} + b_{y,lsg}\mathbb{1}_{f>\kappa}$, $\forall f = f_{\min}^{y,lsg}+1,\ldots,f_{\max}^{y,lsg}$, for $\kappa = f_{\min}+2,\ldots,f_{\max}-2$ (mod-pct(κ))
- Piecewise linear: $\mu_{ynlsg,f} = a_{y,lsg} + b_{y,lsg}(f\mathbb{1}_{f\le\kappa} + \kappa\mathbb{1}_{f>\kappa}) + c_{y,lsg}(f-\kappa)\mathbb{1}_{f>\kappa}$, $\forall f = f_{\min}^{y,lsg}+1,\ldots,f_{\max}^{ynlsg}$, for $\kappa = f_{\min}+3,\ldots,f_{\max}-3$ (mod-plin(κ))

In order to test the classic hypothesis of a constant leaf appearance rate, which corresponds to (mod-ct), we compared the (mod-lin), (mod-pct(κ))), (mod-plin(κ)) models with (mod-ct) for each genotype. First, (mod-ct) was compared to all parametric models and to the complete model using a χ^2-likelihood ratio test ; if all $p > 0.01$, (mod-ct) is selected; otherwise, the parametric model with the highest AIC among the models with $p < 0.01$ is selected.

Monte Carlo EM algorithm for the parametric sub-models. In this section, the indexes y and lsg are omitted. The parametric models we considered are equivalent to imposing a linear constraint:

$$B_q(\mu_{f_{\min}+1},\ldots,\mu_{f_{\max}}) = 0$$

with B_q a $q \times s$-matrix, $s = f_{\max} - f_{\min}$ and $q < s$. The parameter μ^C is unconstrained.

- Constant model (mod-ct):

$$B_q = \begin{pmatrix} 1 & -1 & 0 & \cdot & 0 \\ 1 & 0 & -1 & \cdot & 0 \\ & \cdot & \cdot & \cdot & \\ 1 & 0 & \cdot & 0 & -1 \end{pmatrix}$$

- Linear model: B_q is the $(s-2) \times s$ matrix such that for every $j > 2$

$$\begin{cases} B_q[j-2,1] = j-2 \\ B_q[j-2,2] = 1-j \\ B_q[j-2,j] = 1 \end{cases}$$

and the other coefficients are zero.

- Piecewise constant (mod-pct(κ)): let $\kappa' = \kappa - f_{\min}$, then B_q is the $(s-2) \times s$ matrix with,

$$B_q[j-1,1] = 1, \quad B_q[j-1,j] = -1 \quad \text{if} \quad j = 2,\dots,\kappa'$$
$$B_q[j-1,s] = 1, \quad B_q[j-1,j] = -1 \quad \text{if} \quad j = \kappa',\dots,s-1$$

  and the other coefficients are zero.

- Piecewise linear: let $\kappa' = \kappa - f_{\min}$

  B_q is the $(s-3) \times s$ matrix such that for every $2 < j \leq \kappa'$

$$\begin{cases} B_q[j-2,1] = j-2 \\ B_q[j-2,2] = 1-j \\ B_q[j-2,j] = 1 \end{cases}$$

  and for every $\kappa' + 1 < j \leq s$

$$\begin{cases} B_q[j-2,1] = \kappa-2 \\ B_q[j-2,2] = 1-\kappa \\ B_q[j-2,j] = 1 \\ B_q[j-2,\kappa] = j-\kappa \\ B_q[j-2,\kappa+1] = \kappa-j \end{cases}$$

  and the other coefficients are zero.

The Monte-Carlo E-step is identical to having an unconstrained μ. The M-step corresponds to the maximum likelihood estimation problem from a multivariate Gaussian distributed sample under linear constraints on the mean, which admits an explicit expression as follows (Zoppé et al., 2001). Let B_{s-q} be any matrix such that

$$B = \begin{pmatrix} B_{s-q} \\ B_q \end{pmatrix}$$

is invertible. Then, let

$$S^{(m)} = \frac{1}{nN} \sum_{p=1}^{n} \sum_{r=1}^{N} B(\tilde{\mathbf{Y}}_{p,r}^{(m)} - \bar{\tilde{\mathbf{Y}}}^{(m)})(\tilde{\mathbf{Y}}_{p,r}^{(m)} - \bar{\tilde{\mathbf{Y}}}^{(m)})^t B^t = \begin{pmatrix} S_{s-q,s-q} & S_{s-q,q} \\ S_{q,s-q} & S_{q,q} \end{pmatrix}$$

where $\tilde{\mathbf{Y}}_{p,r}^{(m)} = (Y_{p,r,f}^{(m)})_{f=f_{\min}+1,\dots,f_{\max}}$, $\bar{\tilde{\mathbf{Y}}} = (1/nN)\sum_{p=1}^{n}\sum_{r=1}^{N} \tilde{\mathbf{Y}}_{p,r}$, and $S_{a,b}$ are submatrices of S with dimension $a \times b$. Let $\bar{\tilde{\mathbf{Y}}}_{s-q}$ (resp. $\bar{\tilde{\mathbf{Y}}}_q$) be the subvector of $\bar{\tilde{\mathbf{Y}}}$ with the $s-q$ first (resp. the q last) coordinates of $\bar{\tilde{\mathbf{Y}}}$, then

$$(\mu_{f_{\min}+1}^{(m+1)},\dots,\mu_{f_{\max}}^{(m+1)}) = B^{-1} \begin{pmatrix} \bar{\tilde{\mathbf{Y}}}_{s-q} - S_{q,q}^{-1} S_{s-q,q} \bar{\tilde{\mathbf{Y}}}_q \\ 0 \end{pmatrix}$$

Finally, $(\mu^{C,(m+1)}, \sigma^{C,(m+1)}, \sigma^{(m+1)})$ are given by equation (2).

C Are genotypic group effects due to differences in the average phyllochron?

Phyllochron differences between genotypic groups (genotypes, selection populations or inbred lines) could originate from differences in the average phyllochron (μ^C, μ) and/or in the standard deviation (σ^C, σ). In this subsection, we describe the additional comparisons we performed to check that the genotypic group effect does indeed impact the average phyllochron. In addition to models $M_{j-1,j-1}$ and $M_{j,j}$ (Table 4-A), we considered models $M_{j-1,j-1}^{\sigma}$ where the mean parameters depend on the grouping level $j-1$ and the variance parameters on the grouping level j (Table S3).

The comparison of models $M_{j,j}$ and $M_{j-1,j-1}^{\sigma}$ tests the effect of genotypic groups on the mean parameters $(\mu_{y,lsg,f})$, regardless of its effect on the variance parameters $(\sigma_{y,lsg,f})$. For this comparison, we considered the simple χ^2-likelihood ratio test, without the permutation scheme that would rescale all p-values, as the goal was to compare the p-values with equal and unequal variance

parameters regardless of their absolute values. Results in Table S4 indicate that these comparisons lead to similar p-values as the $M_{j,j}/M_{j-1,j-1}$ comparison. Moreover, the estimates of $(\mu_{y,lsg,f})$ computed under models $M_{j-1,j-1}$ and $M^{\sigma}_{j-1,j-1}$ are very similar (Figure S10). Therefore, the observed genotypic groups effects are due, in a significant part, to differences in the average phyllochron $(\mu^C_{y,lsg}, \mu_{y,lsg,f})$ rather than in the nuisance parameters $(\sigma^C_{y,lsg}, \sigma_{y,lsg,f})$.

D Complementary analyses

Total leaf number

Variations in total leaf number were observed in our dataset (Figure S2). On average, Early genotypes had fewer leaves than Late genotypes from the same genetic background, and this difference was more pronounced in the ancestral line MBS with 16-18 leaves (Early) versus 18-20 leaves (Late). Furthermore, the total leaf number was similar year to year within genotypes, although it was slightly higher in 2014 than in 2015, and in 2015 than in 2016 except for genotype FL317. By construction, the maximum modeled leaf rank $f^{y,lsg}_{\max}$ was correlated with total leaf number, thus the trends observed in the latter were recovered in the former.

Descriptive analysis of cumulated and global instant phyllochrons

Figure S3 shows the estimates of the cumulated phyllochron between sowing and the appearance of leaf 8, as well as the time interval between leaves 8 and 13 in 2014 and 2015:

$$\sum_{f'=9}^{13} \widehat{\mu}_{y,lsg,f'}.$$

We found that both cumulated and instant phyllochrons were strongly impacted by the year, but in opposite directions: while the cumulated phyllochron (Figure S3A) was faster in 2015 than in 2014 (a difference of 3-5 degree-days), the global instant phyllochron (Figure S3B) was faster in 2014 than in 2015 (a difference of 2-3 degree-days). Furthermore differences in instant phyllochrons between years were more marked in the ancestral line MBS (3-7 degree-days) than in F252 (1-2 degree-days).

Assessing the ancestral line effect, we found that the cumulated phyllochron was faster in F252 (28.7 and 24.8 degree-days in 2014 and 2015, respectively) than in MBS (30.2 and 25.7 degree-days in 2014 and 2015, respectively). This means that genotypes from the F252 background produced eight visible leaves more quickly than genotypes from the MBS background. As for the global instant phyllochron, i.e the ATT between leaves 8 and 13, differences between ancestral lines depended on the year of observation. In 2015, it was longer in MBS genotypes (23.7 degree-days) than in F252 genotypes (21.5 degree-days). The opposite was observed in 2014: The global instant phyllochron was 17.8 degree-days in the F252 genotypes and 16.7 degree-days in the MBS genotypes.

No general trend was found between selection populations. However, we observed differences between genotypes from the same selection population. Interestingly, these differences tended to be preserved from one year to the next: FE039 tended to be faster than FE036 (except for the cumulated phyllochron in 2015), FL318 tended to be faster than FL317, and ME049 tended to be faster than ME052 (except for the instant phyllochon in 2014). The two exceptions corresponded to a small difference in absolute value: 0.11 degree-days between the cumulated phyllochrons of FE036 and FE039 in 2015 and 0.39 degree-days between the instant phyllochrons of ME049 and ME052 in 2014, which makes the change of trend between 2014 and 2015 poorly informative. We also observed a difference between the instant and cumulated phyllochron in Late MBS genotypes: ML040 tended to be faster than ML053 up to the leaf 8, and slower between leaves 8 and 13.

Figure S4 provides a more detailed insight into the dynamics of the instant phyllochron by showing the estimates for all leaf ranks where data were available, in the different genotypes. Again, the most striking observation was the strong variation of the temporal trend between years. In 2014 and 2016, the time interval between successive leaves varied moderately (between 3 and 5 degree-days), except between leaves 13 and 14 in the F252 genotypes where the interval was longer, and was globally shorter in 2014 than in 2016 (3.5 versus 4 degree-days on average). In 2015, phyllochrons were more variable throughout the season, ranging between 3 and 7 degree-days, with an increasing and a decreasing phase in MBS genotypes, and a rather constant increase in

F252 genotypes. Note that the particular temporal pattern observed in 2015 (Figure S4) explains the high values of the ATT between leaves 8 and 13 (Figure S3).

Estimation of the phyllochron at the row level

The phyllochron model was implemented separately for each row in the experimental design (a row consisted of plants from a unique genotype). The range of leaf rank $[f^{\min}, f^{\max}]$ for each row was selected such that each rank is observed on at least 6 plants. Despites a smaller threshold, the leaf rank range for the row level phyllochron are smaller than the one at the genotype level due to a smaller number of plants. For each genotype-year combination, Figure S5 displays the complete model and best parametric model at the genotype level, as well as the complete model at the row level for each row consisting of plants from this genotype. Despite variations between rows, the temporal trends detected as significant are recovered in all rows for most genotypes. A few isolated variations were observed but they likely occure from sampling variations, as the number of plants by row is moderate.

Moreover, an alternative procedure to select the best model for each genotype using the row level analysis was implemented, assuming the same parametric model (but with different values of the parameters) for all rows of a genotype. This procedure required to limit the parametric model for each genotype to the models that could be computed with the restricted leaf rank range of all rows. Table S5 shows the χ^2-likelihood ratio p-values and the AIC for each genotype and parametric model. Figures S6 and S7 display the best model selected respectively including or excluding the complete model. Among the parametric models, the selection based on the row level phyllochrons and the genotype level phyllochron lead to very similar temporal trends (Figure S7). Genotype ME049 in 2015 represents a noteworthy exception, but the bilinear model selected at the genotype level was not computable at the row level due to a smaller leaf rank range. For some genotype-year combination, the row level procedure selects the complete model and thus captures small row level variations (Figure S6). But globally, we observe a strong coherence between the model selection at the genotype level, and the selection at the row level with independent plants. This indicates that the analysis of phyllochron dynamics at the genotype level, which is the level of interest from a biological point of view, is reliable.

Principal Components Analysis (PCA) representation : an overview of the outputs of the phyllochron model

PCA was implemented on the set of vectors

$$(\widehat{\mu}^{C(8)}_{y,lsg}, \widehat{\mu}_{y,lsg,9}, \dots, \widehat{\mu}_{y,lsg,14})$$

for all genotypes lsg and years y. For genotypes FE036 and FL317 in 2014, $f^{y,lsg}_{\max} = 13$ thus $\widehat{\mu}_{y,lsg,14}$ could not be estimated and we replaced the two missing values with the average $\widehat{\mu}_{y,lsg,14}$ across all other genotype-year combinations. PCA visualisation allowed for a summarized representation of the findings brought by the phyllochron model, and provided an overview of the global patterns. Thus, it could be used as preliminaries to statistical analyses.

The first axis of Figure S9 (49% of the total inertia) discriminated year 2015 on the right from years 2014 and 2016 on the left. The second axis (21% of the total inertia) discriminated ancestral lines: most F252 genotypes had negative coordinates, whereas MBS genotypes had positive coordinates. No clear discrimination was observed between Early and Late genotypes within each ancestral line.

Furthermore, both the cumulated phyllochron and the instant phyllochron of leaves 9 to 13 were loaded by the components of both axes, and thus were associated with differences between both ancestral lines and years. By contrast, $\widehat{\mu}_{14}$ was mostly associated with ancestral lines. Finally, phyllochron parameters were ordered clockwise by leaf rank in the correlation circle, which indicates that intervals between successive leaves are similar for close leaf ranks.

Descriptive analysis of rainfall, humidity and photosynthetic radiation in year 2015

Since phyllochrons exhibited a particular structure in 2015, we decided to focus on this year to see if we could identify special patterns in the climate variables that might explain the observations. We focused on three variables: rainfall, humidity and photosynthetic radiation (see Figure S13). The

year 2015 was characterized by low rainfall throughout the season and two periods of low humidity and high radiation: the first two weeks of June 2015 and the first week of July 2015. Therefore, 2015 was characterized by the progressive establishment of drought conditions for several weeks which could have contributed to the increase followed by the decrease in phyllochron that year. Thus, the specific phyllochron pattern observed in 2015 (and in last leaf ranks of a few genotypes in 2014) could originate from dry conditions.

References

Botev, Z. and L. Belzile, 2020 Truncatednormal: Truncated multivariate normal and student distributions R package version 2.2.

Zoppé, A., Y.-P. A. Buu, and B. Flury, 2001 Parameter estimation under constraints for multivariate normal distributions with incomplete data. Journal of Educational and Behavioral Statistics **26**: 219–232.

2014

		C	L	PC-9	PC-10	PC-11	PC-12	PC-13	PC-14	PC-15	PC-16	PL-10	PL-11	PL-12	PL-13	PL-14	PL-15	full
FE036	chi2-pv		0.98		0.24	1												0.15
	AIC	343.14	345.09		342.28	345.17												335.32
FE039	chi2-pv		0.074		0.018	0.0038	1						0.91					8.5e-08
	AIC	481.25	478.05		475.19	472.08	483.75						484.7					432.43
FL027	chi2-pv		1.8e-08	3.1e-06	5.5e-10	3.2e-12	8.4e-07	0.29	1			3e-08	9.3e-08	1.8e-08	2.3e-10			1.2e-07
	AIC	681.14	647.52	657.79	640.5	630.2	655.16	680.68	685.59			647.24	649.58	646.25	637.28			627.83
FL317	chi2-pv		3.1e-05		0.0035	0.00037												0.0023
	AIC	445.15	426.42		435.84	431.34												424.17
FL318	chi2-pv		0.18		0.29	1	0.03						0.3					0.0046
	AIC	551.56	550.13		551.11	554.39	546.55						551.89					531.46
ME049	chi2-pv		0.012		9.6e-05	0.0019	0.12	0.67	0.81				2.5e-05	1.2e-05	0.00027			0.037
	AIC	401	394.2		384.5	390.5	398.82	402.19	402.58				380.99	379.45	385.97			386.25
ME052	chi2-pv		1		0.49	1	0.87	1	0.75				0.63	0.75	1			0.81
	AIC	468.4	470.47		468.99	470.54	470.12	470.86	469.82				470.68	471.18	472.53			470.59
ML040	chi2-pv		0.0032		0.23	0.01	0.027	0.0034	0.00089	0.018			0.0086	0.0089	0.0074	0.0079		0.13
	AIC	545.53	536.02		544.63	538.4	540.34	536.14	533.48	539.47			537.86	537.94	537.55	537.69		535.67
ML053	chi2-pv		0.82		0.15	0.48	0.9	0.97	0.91	1	0.65		0.54	0.45	0.56	0.66	0.61	0.79
	AIC	380.68	382.28		378.91	381.2	382.46	382.61	382.5	382.74	381.82		382.5	382.01	382.63	383.06	382.87	383.12

2015

		C	L	PC-9	PC-10	PC-11	PC-12	PC-13	PC-14	PC-15	PC-16	PL-10	PL-11	PL-12	PL-13	PL-14	PL-15	full
FE036	chi2-pv		0		3.4e-15	0	2.7e-15						<2.e-16					<2.e-16
	AIC	1109.7	1002.9		1045.1	1010.6	1044.5						1002.6					999.75
FE039	chi2-pv		<2.e-16		1.1e-16	4.4e-16	1.3e-12						<2.e-16					2e-14
	AIC	1035.1	944.17		962.71	966.32	982.29						945.11					950.6
FL027	chi2-pv		3.2e-08		1.4e-12	5.3e-07	0.055	0.92	0.037				1.3e-12	7.6e-14	2.4e-10			7.8e-11
	AIC	903.6	871.09		851.05	876.68	899.79	905.42	899.02				849.28	843.43	859.86			833.71
FL317	chi2-pv		<2.e-16		4.2e-13	7.6e-12	3.8e-10						1.1e-16					2.7e-11
	AIC	728.52	654.09		673.53	679.32	687.11						655.6					660.55
FL318	chi2-pv		<2.e-16		<2.e-16	2.4e-11	1.5e-08						<2.e-16					<2.e-16
	AIC	1171.1	1070.6		1074.5	1124.2	1137.1						1052.3					1057.1
ME049	chi2-pv		0.00093		0	1.8e-06	1	8.1e-06	7.4e-06				<2.e-16	<2.e-16	<2.e-16			<2.e-16
	AIC	1518.2	1506.2		1418.8	1493.8	1521.2	1496.8	1496.6				1393.6	1347.3	1408			1281.5
ME052	chi2-pv		1	4.4e-06	0.86	1	0.37	0.12	1			6.6e-12	<2.e-16	<2.e-16	6e-08			<2.e-16
	AIC	1299.7	1309	1277	1301.4	1308.1	1299.7	1297.5	1304.9			1248.6	1188.8	1207.5	1267.2			1134.7
ML040	chi2-pv		1		0.21	1	1	0.004	5e-04	0.17			<2.e-16	<2.e-16	1.4e-10	0.0026		<2.e-16
	AIC	976.22	1004.6		975.14	1001.4	978.66	967.18	963.04	974.67			883.88	885.6	931.4	965.98		839.69
ML053	chi2-pv		1		0.0017	1	0.32	0.0032	0.019	0.74	1		3.6e-15	3.1e-11	1.7e-05	0.13	1	6.1e-12
	AIC	722.75	726.23		712.02	725.73	722.49	713.27	716.86	724.14	724.85		656.38	674.83	701.99	721.09	728.88	642.57

2016

		C	L	PC-9	PC-10	PC-11	PC-12	PC-13	PC-14	PL-10	PL-11	PL-12	PL-13	full
ME052	chi2-pv		0.024	0.45	0.016	0.00079	6e-05	0.51		0.0062	0.024	0.058		4.9e-05
	AIC	2130.4	2124.9	2130.8	2124.1	2118.1	2112.9	2131		2122	2125	2126.9		2094.8
ML040	chi2-pv		0.0052	0.00038	0.2	0.069	0.18	0.28	0.043	0.00066	0.0089	0.013	0.015	0.0073
	AIC	1710.5	1701.9	1696.7	1709.2	1707.1	1709	1709.9	1706.2	1697.3	1702.8	1703.6	1704	1689.2

Table S1: **Comparison of parametric submodels**. p-value of the χ^2-likelihood ratio test between the constant model (null hypothesis) and an alternative model and AIC. Parametric models for $f \mapsto \mu_f$: 'C'=constant, 'L' = linear, 'PC-f'=piecewise constant with cut in leaf rank f, 'PL-f'=piecewise linear with cut in leaf rank f, 'full'= general model (no assumption on the structure of μ_f. The model which minimises each criterion is highlighted.

Ancestral line effect

	M_{11}/M_{00} line effect on (C+I)	M_{11}/M_{10} line effect on I	M_{10}/M_{00} line effect on C
2014	$<$ e-16	3.20e-13	1.70e-11
2015	$<$ e-16	$<$ e-16	2.60e-12

Selection effect

	M_{22}/M_{11} selection effect on (C+I)	M_{22}/M_{21} selection effect on I	M_{21}/M_{11} selection effect on C
2014.F	2.00e-12	2.60e-12	8.60e-02
2014.M	4.20e-02	7.20e-02	7.20e-02
2015.F	3.00e-03	1.70e-03	9.00e-01
2015.M	1.30e-05	2.90e-03	6.00e-05

Residual genotypic effect

	M_{33}/M_{22} genotype effect on (C+I)	M_{33}/M_{32} genotype effect on I	M_{32}/M_{22} genotyep effect on C
2014.Fearly	6.5e-05	1.1e-03	2.5e-03
2014.Flate	5.6e-12	2.1e-10	1.5e-03
2014.Mearly	6.0e-02	7.6e-02	1.4e-01
2014.Mlate	3.8e-05	1.8e-03	5.3e-04
2015.Fearly	4.9e-04	2.6e-04	9.4e-01
2015.Flate	3.7e-09	7.3e-07	1.4e-04
2015.Mearly	<e-16	2.6e-10	4.9e-13
2015.Mlate	1.5e-04	1.0e-02	2.6e-04

Table S2: χ^2**-likelihood ratio** p**-values for ancestral line, selection and genotypic effects.** For each grouping effect, models $M_{j-1,j-1}$, $M_{j-1,j}$, and $M_{j,j}$ were compared (see Table 4-A).

Model	μ^C parameters	μ parameters
M^σ_{00}	$\forall(l,s,g),\quad (\mu^C_{lsg},\sigma^C_{lsg}) = (\mu^C,\sigma^C_l)$	$\forall(l,s,g),\quad (\mu_{lsg},\sigma_{lsg}) = (\mu,\sigma_l)$
M^σ_{11}	$\forall(s,g),\quad (\mu^C_{lsg},\sigma^C_{lsg}) = (\mu^C_l,\sigma^C_{ls})$	$\forall(s,g),\quad (\mu_{lsg},\sigma_{lsg}) = (\mu_l,\sigma_{ls})$
M^σ_{22}	$\forall(g),(\mu^C_{lsg},\sigma^C_{lsg}) = (\mu^C_{ls},\sigma^C_{lsg})$	$(\mu_{lsg},\sigma_{ls}) = (\mu_{lsg},\sigma_{lsg})$

Table S3: Models in which the mean parameters depend on grouping level $j-1$ and the variance parameters on grouping level j.

2014

	$M_{j,j}/M_{j-1,j-1}$	$M_{j,j}/M^\sigma_{j-1,j-1}$
all	<2.e-16	7.3e-15
F	2.00e-12	1.8e-09
M	4.20e-02	7.2e-02
Fearly	6.60e-05	3.6e-05
Flate	5.70e-12	1.1e-9
Mearly	6.00e-02	7.3e-02
Mlate	3.80e-05	6.3e-06

2015

	$M_{j,j}/M_{j-1,j-1}$	$M_{j,j}/M^\sigma_{j-1,j-1}$
all	<2.e-16	<2.e-16
F	3.0e-03	1.7e-02
M	1.3e-05	2.0e-04
Fearly	4.9e-04	6.8e-05
Flate	3.7e-09	9.4e-06
Mearly	<2.e-16	<2.e-16
Mlate	1.5e-04	2.0e-05

Table S4: χ^2-likelihood ratio p-value for the genotypic group effect on the mean and variance of the phyllochron ($M_{j,j}/M_{j-1,j-1}$) and on the average phyllochron μ only ($M_{j,j}/M^\sigma_{j-1,j-1}$).

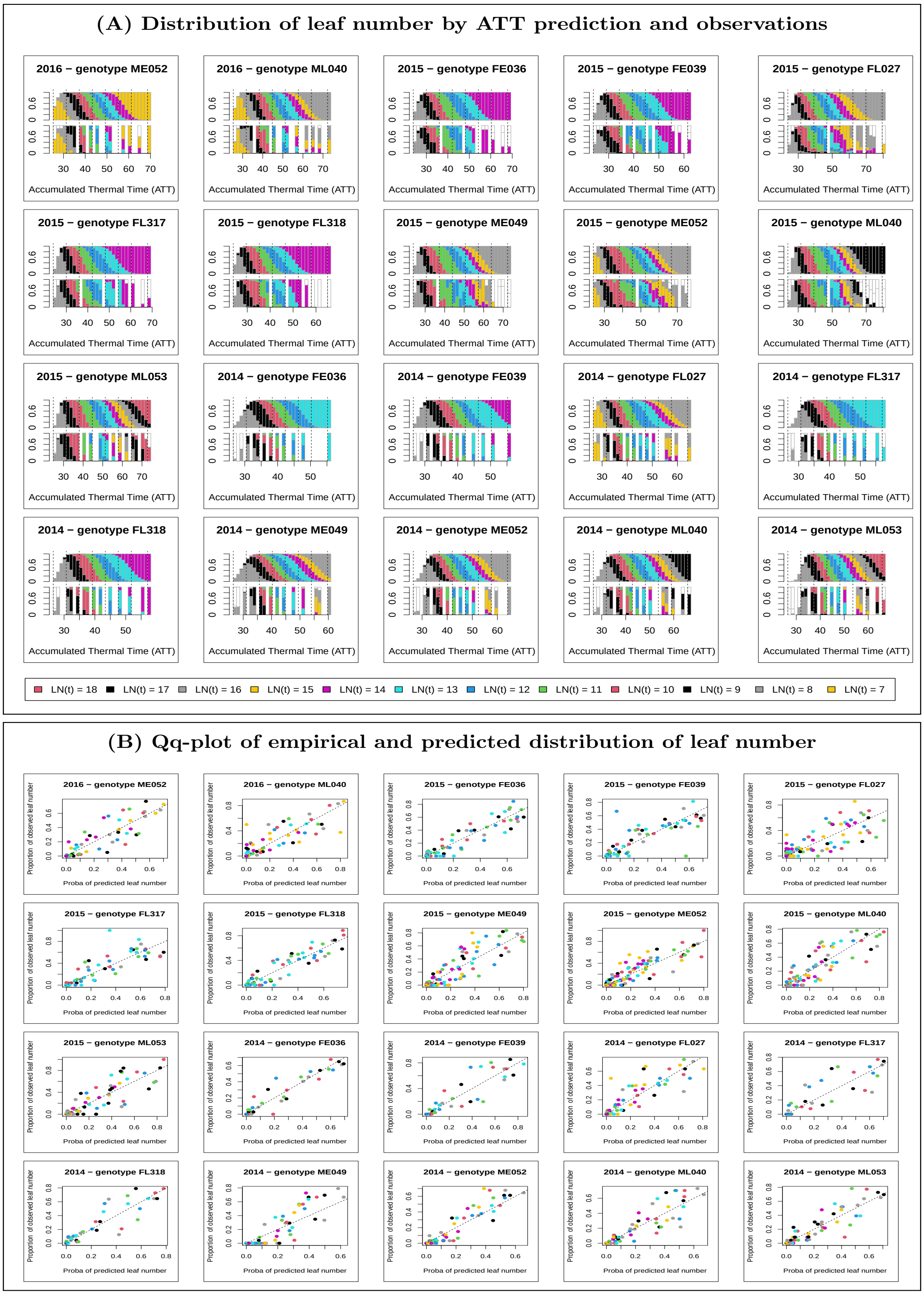

Figure S1: . **Predicted and observed leaf number distribution**. (A) For each genotype-year combination, the top graph shows the distribution of the number of visible leaves $LN(t)$ predicted by the model for t in each ATT (Accumulated Thermal Time) interval. The bottom graph provides the distribution of the observed $LN(t)$ for all measurements of plants of the genotype-year within each ATT interval. Each color corresponds to a leaf rank in the modeled range of leaf ranks. Non-modeled leaf ranks are in white. Vertical dashed lines were added to facilitate the visual comparison.(B) For each genotype and year, each point represents the proportion of observations of a given leaf rank in a given ATT interval as a function of its predicted counterpart. Colors correspond to leaf ranks, and dashed line to $y = x$.

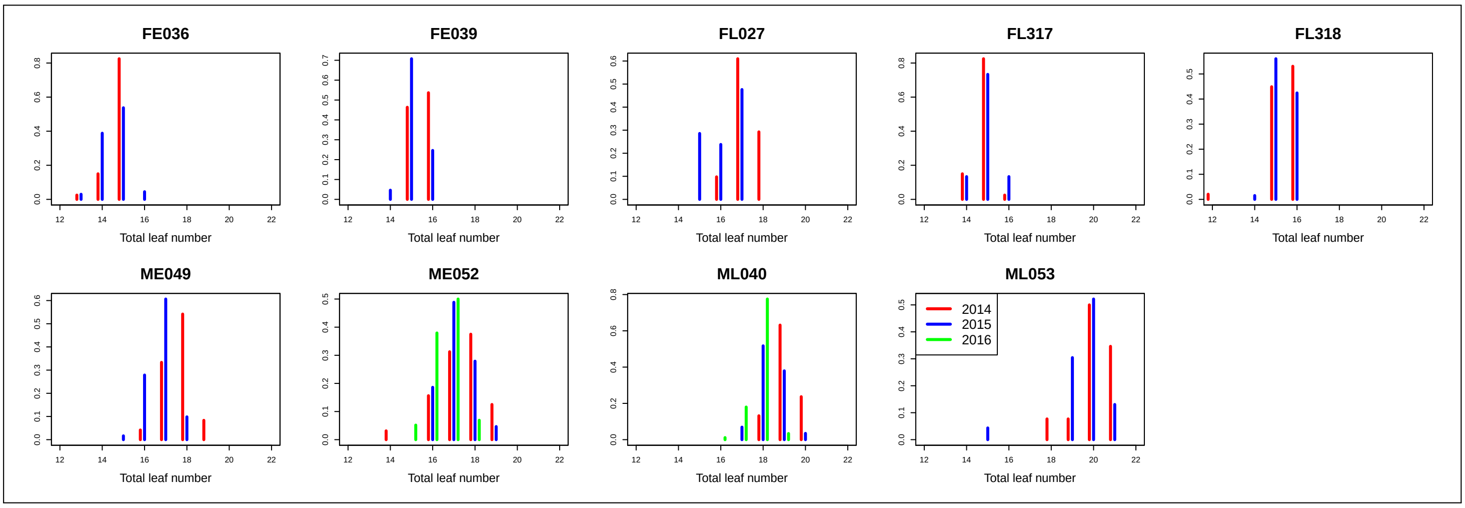

Figure S2: **Distribution of the total leaf number for all plants by genotype and by year.**

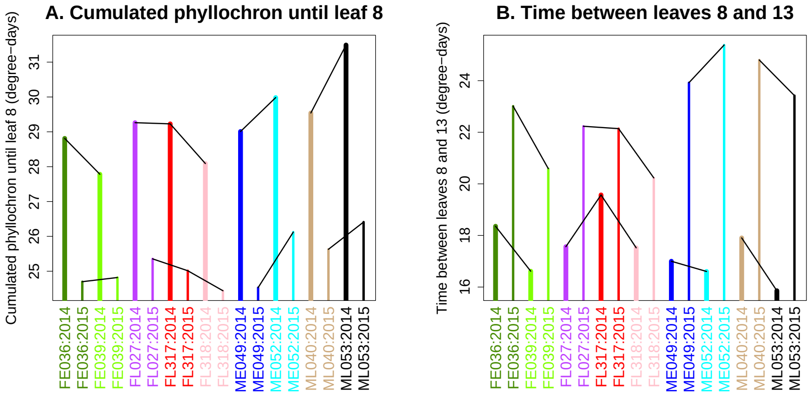

Figure S3: **Cumulated and instant phyllochrons. A.** Accumulated thermal time (ATT) in degree-days to reach eight visible leaves. **B.** Time interval in degree-days between the appearance of leaves eight and 13. Genotypes from the same selection population observed in the same year are joined with a straight line. Thin bars correspond to year 2015 and thick bars to 2014.

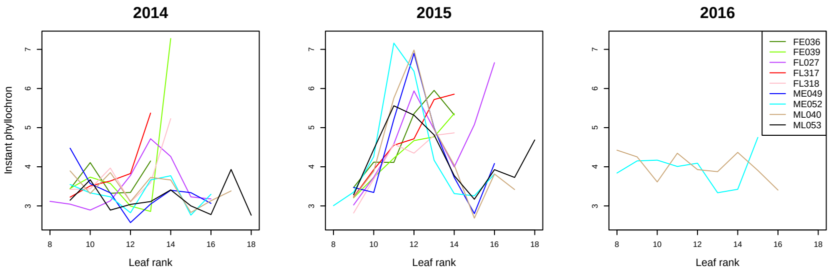

Figure S4: **Estimates of instant phyllochrons with the full models.** Each plot corresponds to a year; genotypes are color coded; lines join estimates $(\widehat{\mu}_{y,lsg,f})_{f=f^{y,lsg}_{\min}+1,\dots,f^{y,lsg}_{\max}}$ from the same genotype lsg at successive leaf ranks.

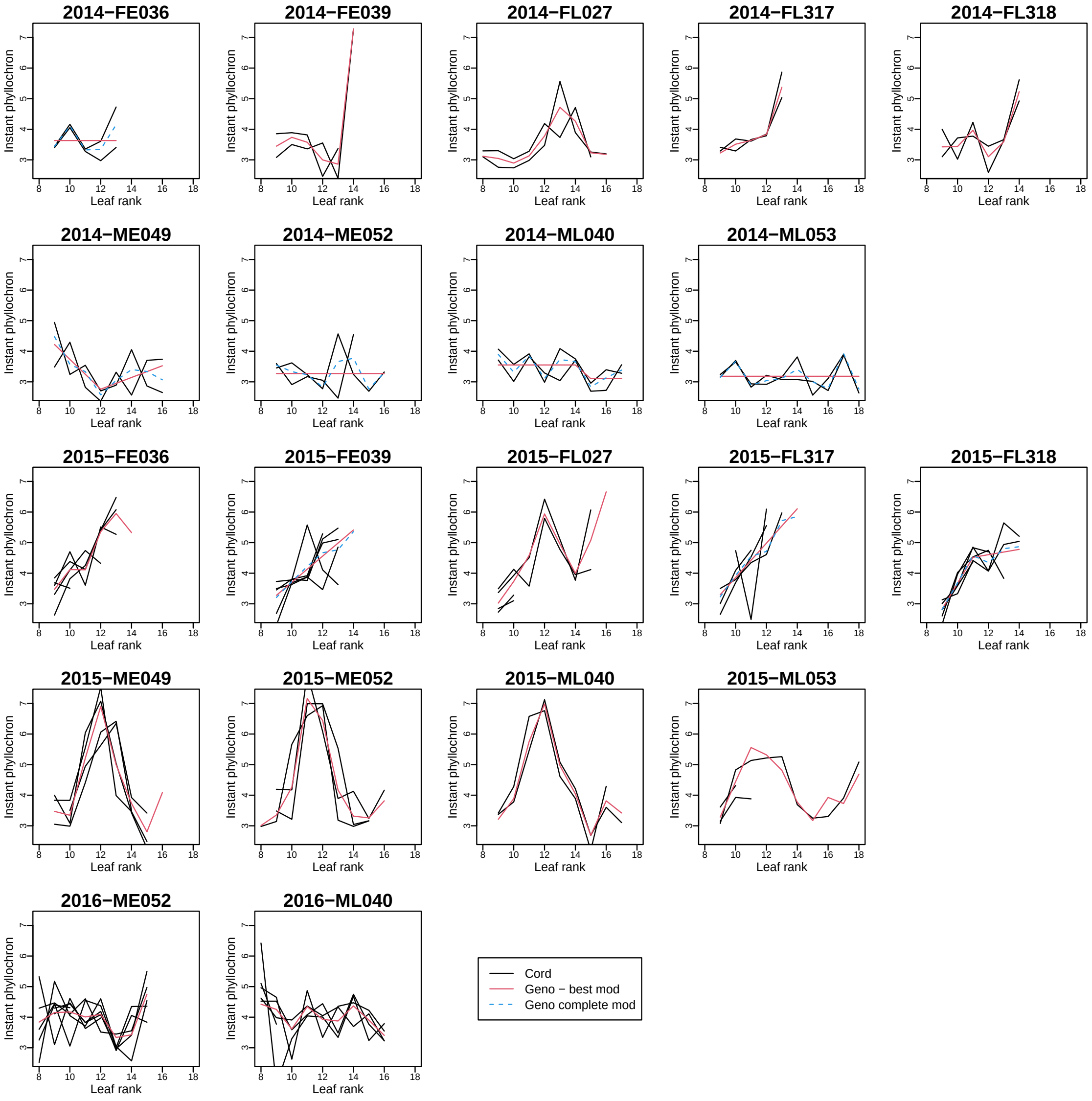

Figure S5: For each genotype-year combination, phyllochron estimates at the row level for each row (solid black line), phyllochron estimate at the genotype level for the best model (red solid line) and for the complete model (dashed blue line) when the best model was a parametric model.

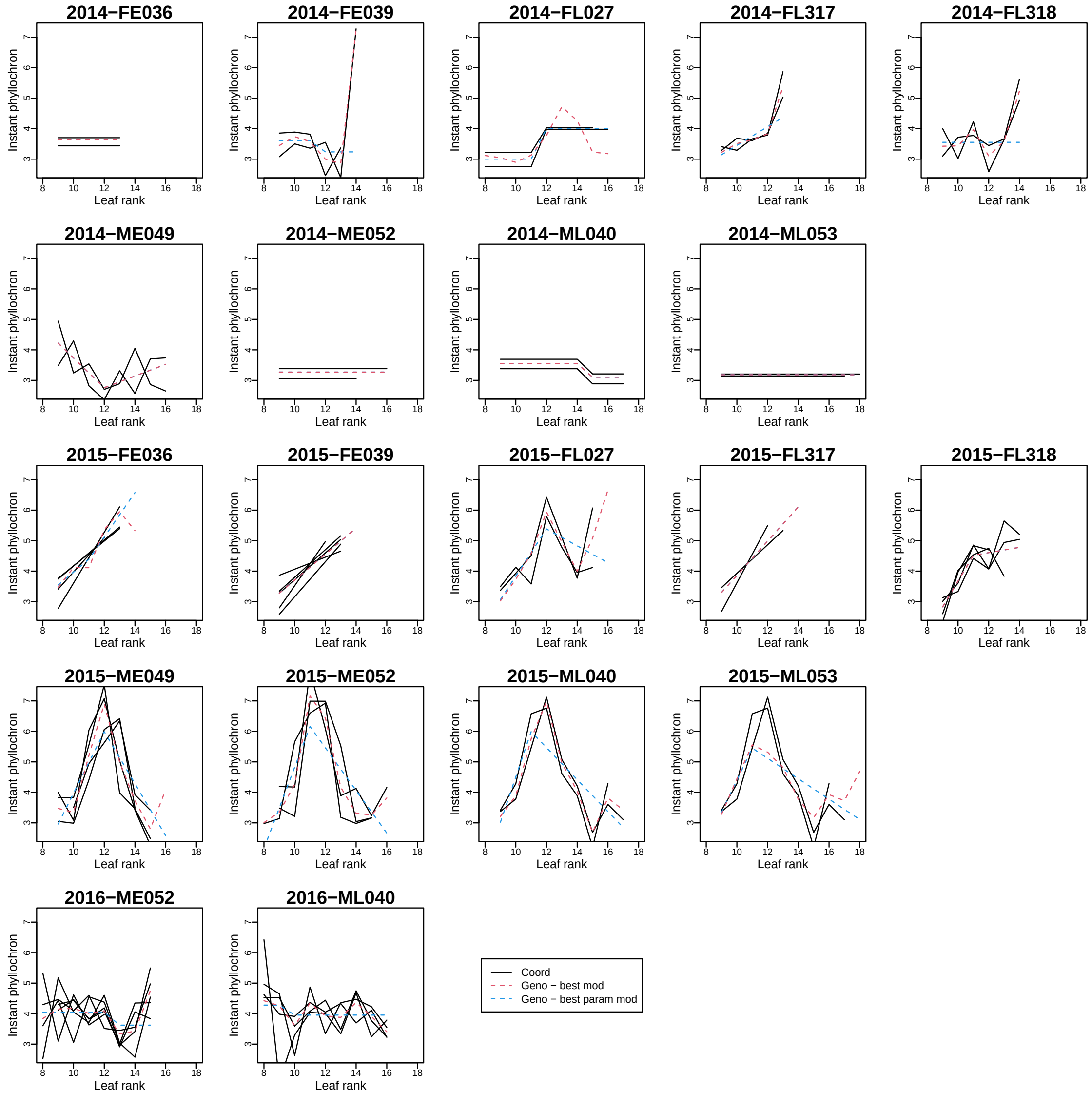

Figure S6: For each genotype-year combination, phyllochron estimates of each row (black) for the best model (including the complete model) selected by the procedure at the row level, and best genotype level phyllochron estimates selected with the procedure at the genotype level (red), including or excluding the complete model

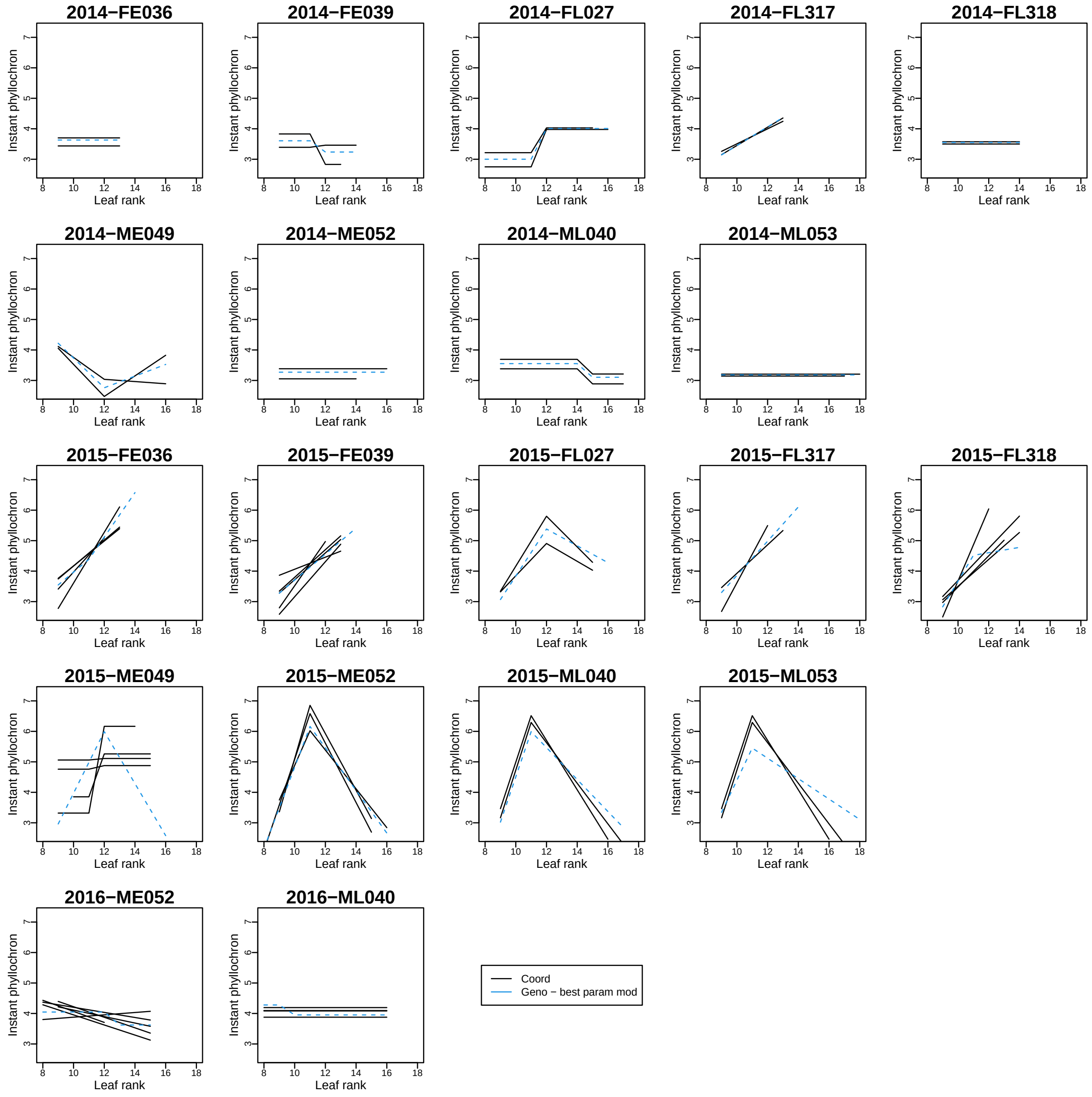

Figure S7: For each genotype-year combination, phyllochron estimates of each row (black) for the best parametric model selected by the procedure at the row level, and best parametric genotype level phyllochron estimates selected with the procedure at the genotype level (red),

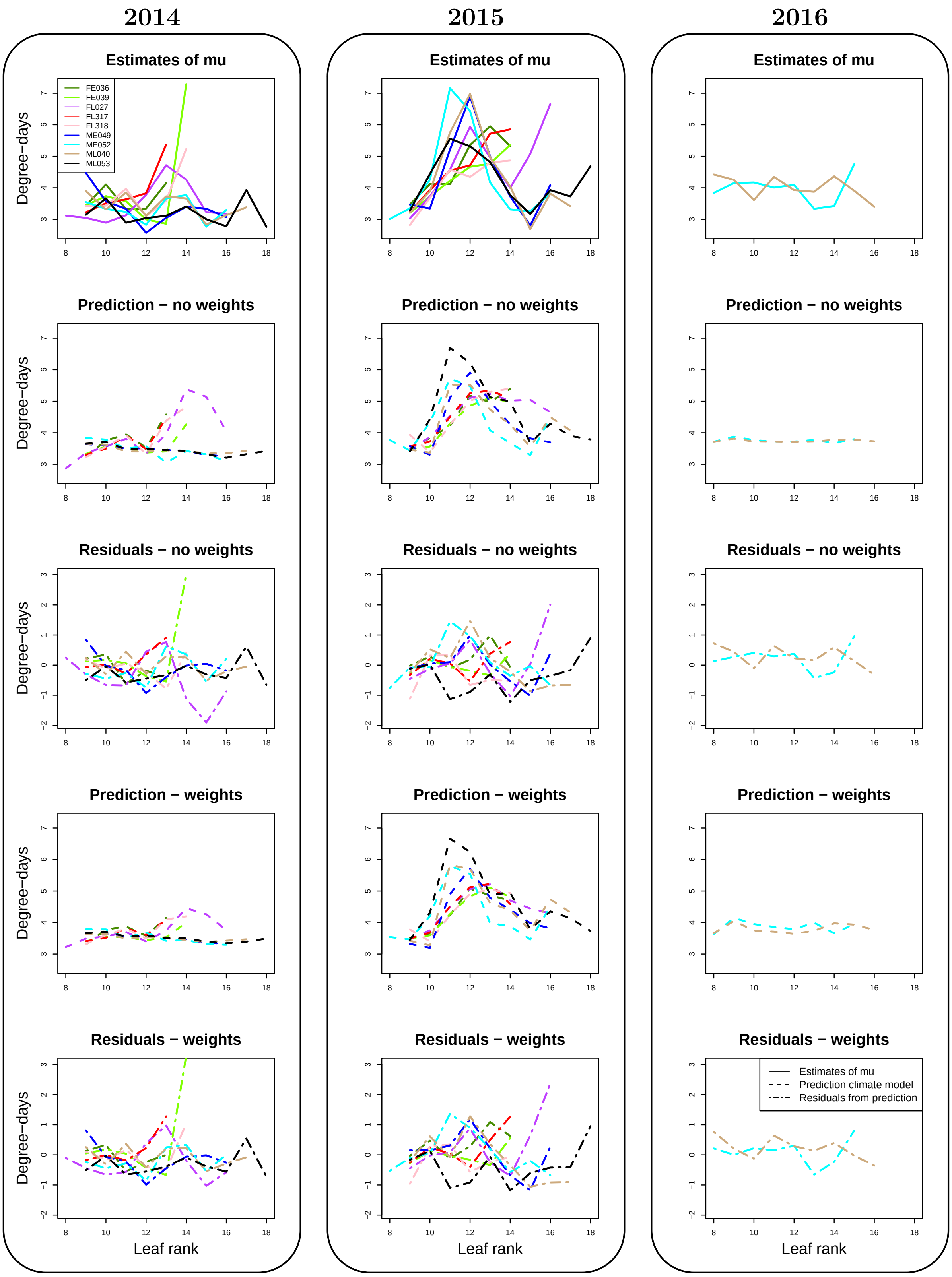

Figure S8: . **Predicted values and residuals of the climate model**. The climate model is implemented using the phyllochron estimates with the complete model. Each plot corresponds to a year and each color to a genotype. Estimates of $(\mu_{y,lsg,f})$ as a function of leaf rank are shown in row 1; Rows 2 and 4 show the predicted values of $\widehat{\mu}_{y,lsg,f}$ with the climate model (3 and 4) in a cross-validation framework, for the unweighted and weighted procedure respectively. Rows 3 and 5 shows the residuals of the prediction i.e. the difference between estimate and prediction curves.

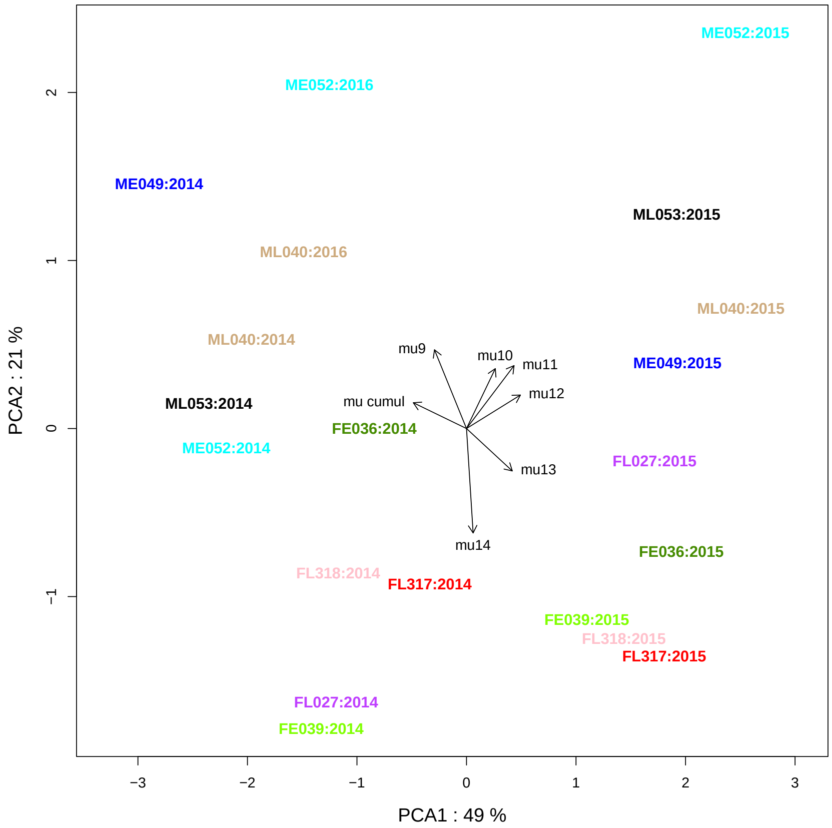

Figure S9: **Principal Component Analysis of seasonal variations in phyllochron values.** Variables are the mean at the genotype level of the time $\widehat{\mu}^{C(8)}_{y,lsg,8}$ between sowing and the appearance of leaf eight and the instant phyllochrons $(\widehat{\mu}_{y,lsg,9}, \ldots, \widehat{\mu}_{y,lsg,14})$ between leaf ranks nine and 14. Individuals are the genotype-year combinations. Individuals are represented on the first two axes of the PCA and colors correspond to the genotypes. Correlations between variables and PCA axes are represented by the black arrows using an arbitrary scale.

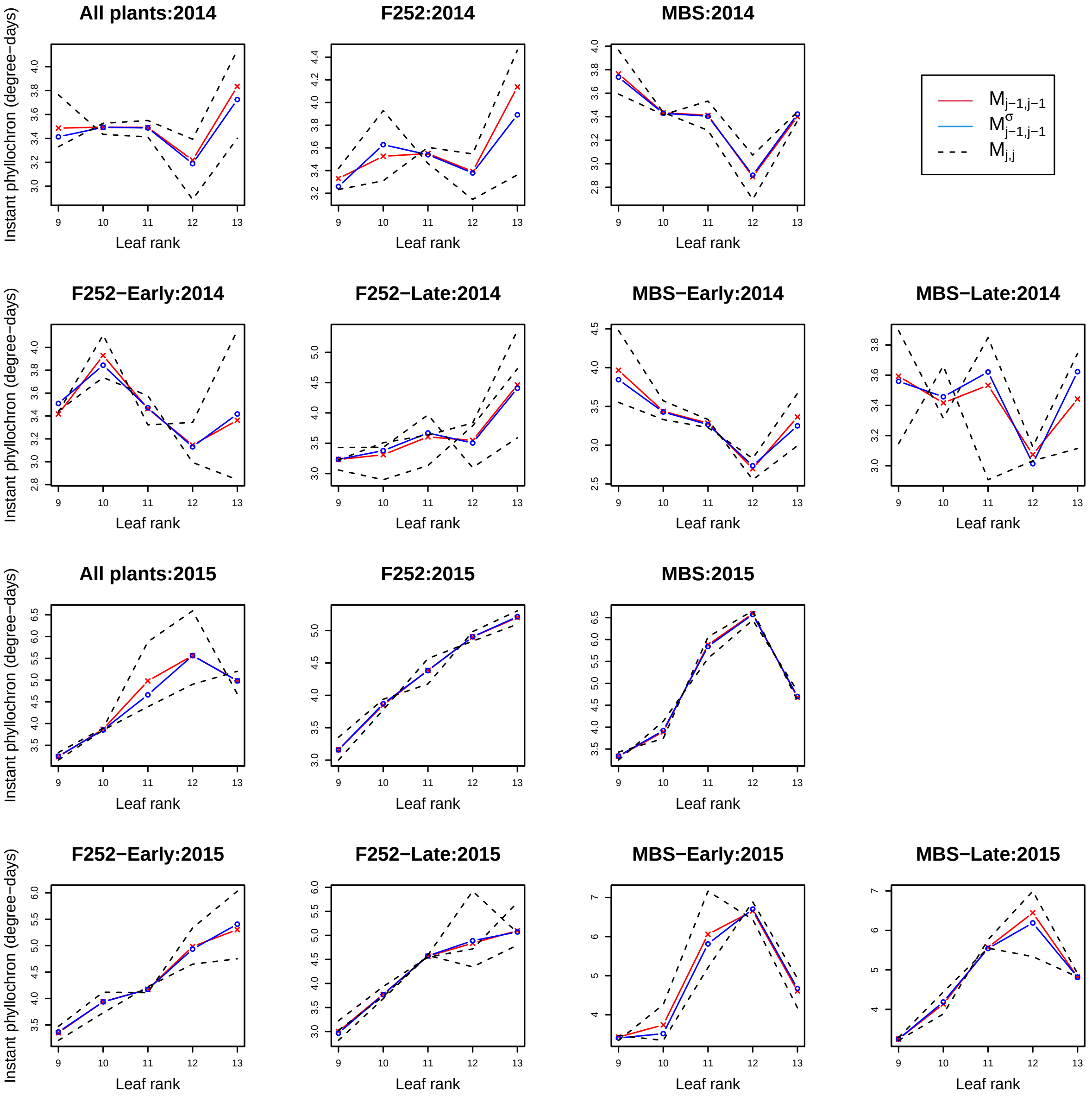

Figure S10: The estimates of the instant phyllochron for models $M_{j-1,j-1}$ (($\mu^C, \mu, \sigma^C, \sigma$) depend on grouping level $j-1$ in red), $M_{j,j}$ (($\mu^C, \mu, \sigma^C, \sigma$) depend on grouping level j in dashed lines) and $M^{\sigma}_{j-1,j-1}$ ((μ^C, μ) depend on grouping level $j-1$ and (σ^C, σ) depend on grouping level j in blue). Models are described in Tables 4-A and S3. Rows 1 and 2 correspond to year 2014, and rows 3 and 4 to 2015. For both years, plots entitled "All plants" correspond to $j = 1$, "F252" and "MBS" to $j = 2$, and "F252-Early", "F252-Late", "MBS-Early" and "MBS-Late" to $j = 3$.

2014

	C	L	PC-10	PC-11	PC-9	PC-12	PC-13	PL-10	PL-11	PL-12	PC-14	PL-13	PC-15	PL-14	full
FE036 chi2-pv		0.215	0.055	0.123											0.0175
FE036 AIC	365	366	363	365											359
FE039 chi2-pv		0.000993	0.00325	5.38e-05											3.01e-08
FE039 AIC	475	466	468	460											436
FL027 chi2-pv		4.07e-09	1.56e-09	1.2e-12	0.000272	2.8e-08	0.374	1.24e-08	5.96e-08	5.44e-08					5.89e-10
FL027 AIC	682	648	646	632	670	652	684	648	651	651					633
FL317 chi2-pv		0.000303	0.00882	0.0005											0.00162
FL317 AIC	448	436	442	437											435
FL318 chi2-pv		0.0112	0.124	0.0314		0.0371			0.0313						2.33e-07
FL318 AIC	565	560	565	562		562			562						531
ME049 chi2-pv		0.00995	0.00107	0.00338		0.0789	0.663		0.00056	0.000184	0.132	0.00189			0.000281
ME049 AIC	416	411	406	408		415	419		404	402	416	407			401
ME052 chi2-pv		1	0.632	1		1			0.667						0.013
ME052 AIC	471	475	474	475		475			476						466
ML040 chi2-pv		0.000791	0.139	0.00637		0.0124	0.000601		0.00452	0.00482	0.000102	0.003	0.00347	0.00426	0.0042
ML040 AIC	566	556	567	560		562	556		559	560	552	558	559	559	561
ML053 chi2-pv		0.647	0.116	0.326		0.626	0.838		0.651	0.548	0.686	0.605	0.951	0.614	0.485
ML053 AIC	397	400	396	398		400	400		402	402	400	402	401	402	412

2015

	C	L	PC-10	PC-11	PC-12	PC-13	PL-11	PL-12	PC-14	PL-13	full
FE036 chi2-pv		2.22e-16	1.97e-08								5.31e-13
FE036 AIC	913	842	879								843
FE039 chi2-pv		2.64e-12	1.47e-08								1.06e-09
FE039 AIC	975	922	940								922
FL027 chi2-pv		0.012	0.000153	0.000287	1	1	0.000228	1.59e-07			5.36e-08
FL027 AIC	524	519	511	512	530	530	510	495			486
FL317 chi2-pv		2.71e-13	1.58e-10								1.47e-09
FL317 AIC	435	381	394								389
FL318 chi2-pv		<2.e-16	<2.e-16								<2.e-16
FL318 AIC	1.03e+03	934	943								933
ME049 chi2-pv		8.31e-09		3.08e-10	8e-08						<2.e-16
ME049 AIC	1.35e+03	1.32e+03		1.31e+03	1.32e+03						1.07e+03
ME052 chi2-pv		4.49e-07	<2.e-16	0.0019	9.26e-09	1.3e-10	<2.e-16	<2.e-16			<2.e-16
ME052 AIC	1.05e+03	1.02e+03	960	1.04e+03	1.01e+03	1.01e+03	834	847			774
ML040 chi2-pv		0.00857	2.9e-05	0.0959	0.00138	1.79e-10	<2.e-16	<2.e-16	1.96e-08	1.59e-11	<2.e-16
ML040 AIC	781	775	764	780	772	740	682	684	749	732	645
ML053 chi2-pv		0.00857	2.9e-05	0.0959	0.00138	1.79e-10	<2.e-16	<2.e-16	1.96e-08	1.59e-11	<2.e-16
ML053 AIC	781	775	764	780	772	740	682	684	749	732	645

2016

	C	L	PC-9	PC-10	PC-11	PC-12	PC-13	PC-14	PL-10	PL-11	PL-12	PL-13	full
ME052 chi2-pv		9.28e-08	1.89e-07										4.996e-15
ME052 AIC	1546	1514	1516										1462
ML040 chi2-pv		0.129	0.865	0.0567	0.475	0.377	0.537	0.152	0.132	0.466	0.436	0.393	0.002712
ML040 AIC	1191	1192	1198	1190	1196	1195	1196	1192	1195	1199	1199	1199	1191

Table S5: **Comparison of parametric submodels at the row level**. p-value of the χ^2-likelihood ratio test between the constant model (null hypothesis) and an alternative model and AIC, where the alternative hypothesis assumes that the phyllochron of all coordinates of a given genotype follow the same parametric sub-model. Parametric models for $f \mapsto \mu_f$: 'C'=constant, 'L' = linear, 'PC-f'=piecewise constant with cut in leaf rank f, 'PL-f'=piecewise linear with cut in leaf rank f, 'full'= general model (no assumption on the structure of μ_f.

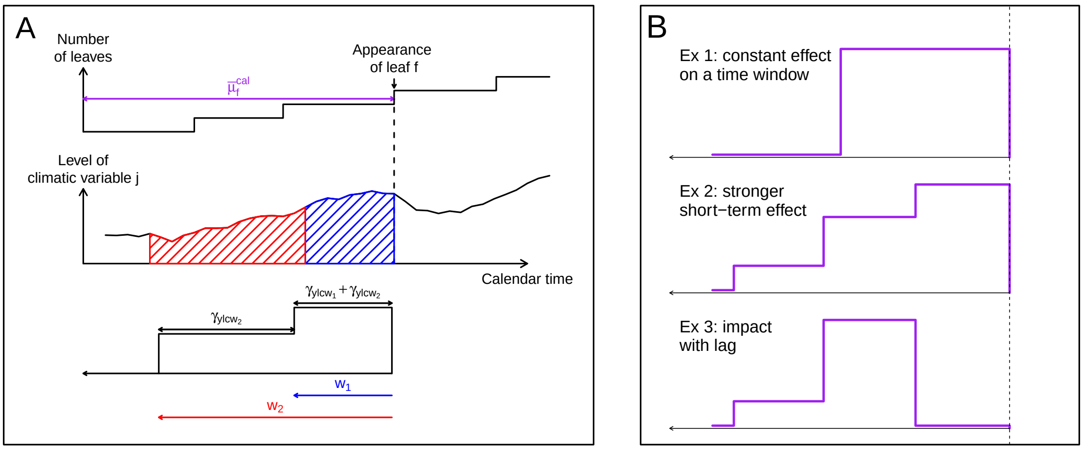

Figure S11: **Climate model.** (A) The top graph shows the average phyllochron process for a given genotype and year in calendar time; the graph below gives the daily values of a climatic variable; the piecewise constant function at the bottom corresponds to the weights applied to the value of the climate variable (see model (4)). On this example, vector $(\gamma_{y,l,c,w})_w$ has two non-zero coefficients for $w = w_1$ and $w = w_2$, therefore the impact of the climate variable on $\mu_{y,lsg,f}$ is equal to the sum of the daily values of the climate variable over the time interval $[\mu_{y,lsg}^{C(f),cal} - w_2, \mu_{y,lsg}^{C(f),cal} - 1]$, weighted by $\gamma_{y,l,c,w_1} + \gamma_{y,l,c,w_2}$ on $[\mu_{y,lsg}^{C(f),cal} - w_1, \mu_{y,lsg}^{C(f),cal} - 1]$ and by γ_{y,l,c,w_2} on $[\mu_{y,lsg}^{C(f),cal} - w_2, \mu_{y,lsg}^{C(f),cal} - w_1 - 1]$. (B) Examples of behaviors that can be modeled by (4) with three non-zero coefficients in $(\gamma_{y,l,c,w})_w$. In the first example, the time between the appearance of successive leaves is assumed to result from the values of the climate variable on a certain time window before the leaf appearance, with the same impact on the whole window. On the second example, the impact of the covariate is assumed to be greater for the value close to the leaf appearance time. In the last example, the impact of the climatic variable is assumed to experience a lag.

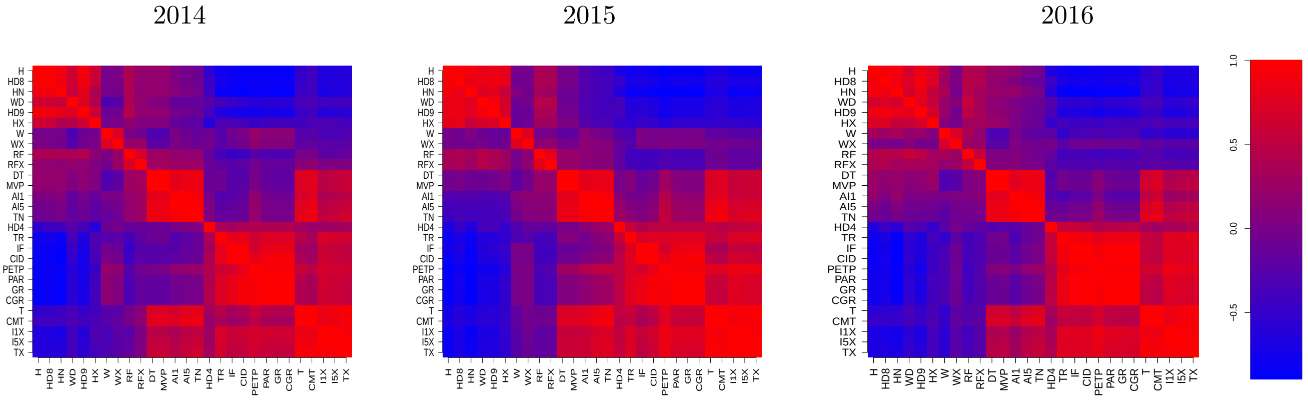

Figure S12: **Pearson correlation between climate variables in years 2014, 2015 and 2016**. The order of the variables on the x and y axis is identical in the three plots. Climate variables are described in Table 2.

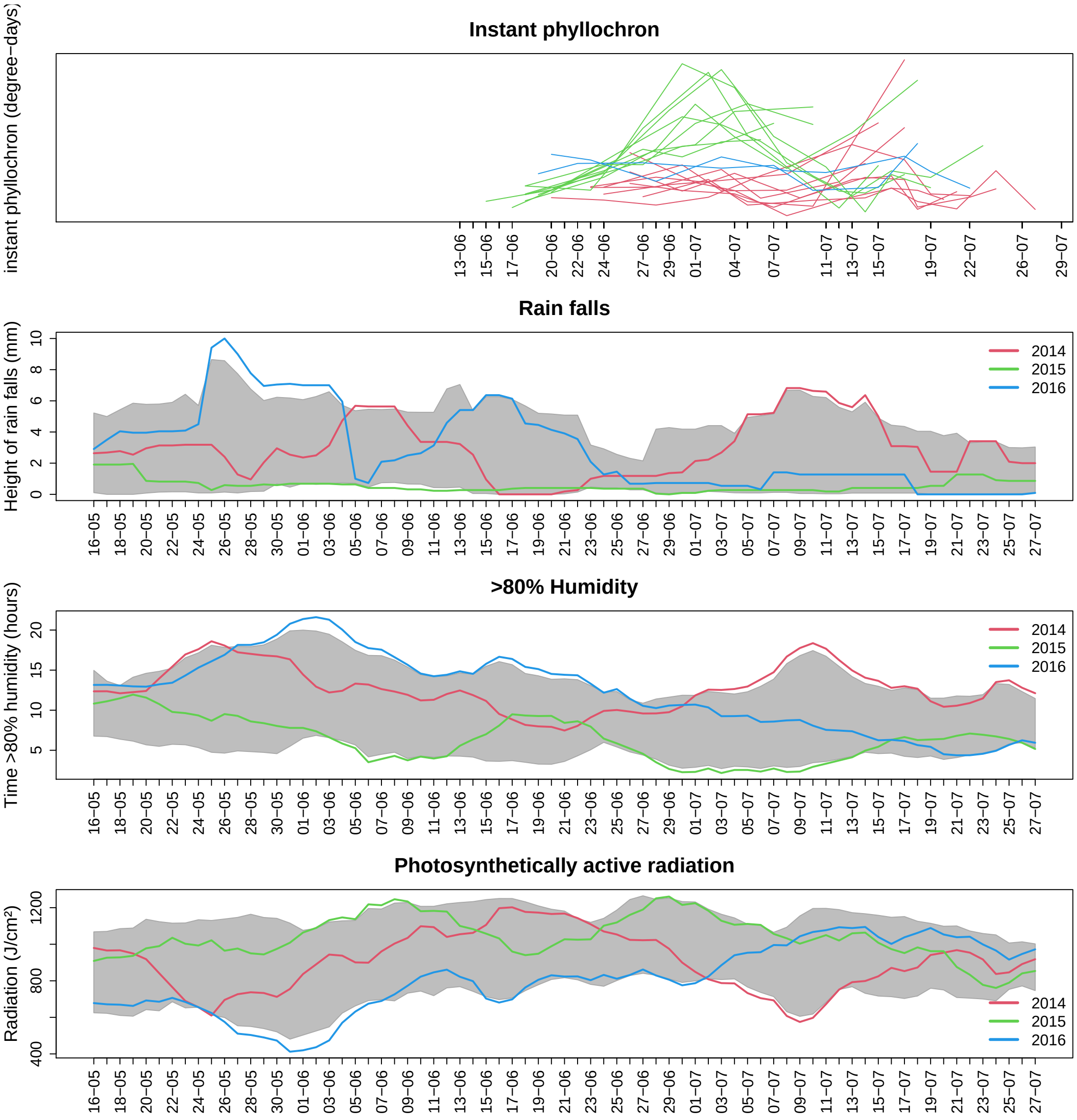

Figure S13: **Instant phyllochron and climate variables recorded in Saclay during the May-July period from 2009 to 2017**. The top graph shows the instant phyllochron of each genotype during the three years of experimentation as a function of calendar time. The following graphs show the climate trends from 2009 to 2017, as well as the specific curves for years 2014, 2015 and 2016 for three variables related to rainfall, humidity and photosynthetic radiation. Daily records of rain fall height, the number of hours with >80% humidity and photosynthetically active radiation were smoothed on a -5/+5day window. Gray areas represent the range of each climate variables for years from 2009 to 2017, excluding for each day the minimal and maximal values. Specific patterns of years from 2014 to 2016 are color-coded (2014:red, 2015:green 2016:blue)

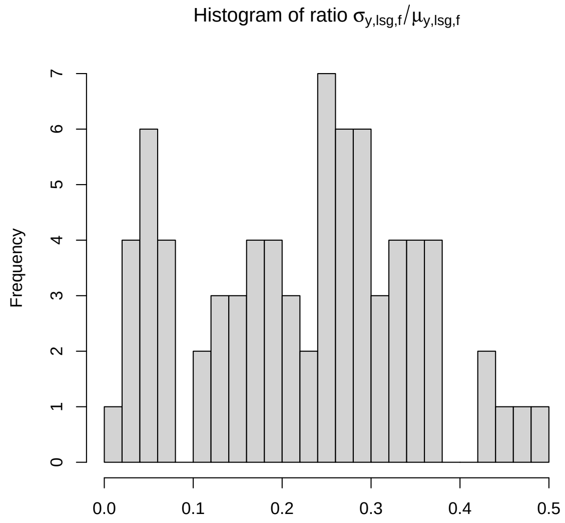

Figure S14: Histogram of the ratio between estimates of the standard deviation $\sigma_{y,lsg,f}$ and the mean $\mu_{y,lsg,f}$ for all years, all genotypes and all leaf ranks. The maximum value of the ratio is 0.5, which corresponds to a probability of 0.02 to be negative for the normal distribution with these parameters. But this probability is much lower for most genotype-year combinations (e.g. $p = 3.10^{-7}$ for $\sigma_{y,lsg,f}/\mu_{y,lsg,f} = 0.2$)
